# A new Miocene pinniped *Allodesmus* (Mammalia: Carnivora) from Hokkaido, northern Japan

**DOI:** 10.1098/rsos.172440

**Published:** 2018-05-16

**Authors:** Wataru Tonomori, Hiroshi Sawamura, Tamaki Sato, Naoki Kohno

**Affiliations:** 1 Graduate School of Life and Environmental Sciences, University of Tsukuba, Ibaraki, Japan; 2 Ashoro Museum of Paleontology, Hokkaido, Japan; 3 Department of Astronomy and Earth Sciences, Tokyo Gakugei University, Tokyo, Japan; 4 Department of Geology and Paleontology, National Museum of Nature and Science, Ibaraki, Japan

**Keywords:** Pinnipedia, *Allodesmus*, middle Miocene, western North Pacific

## Abstract

A nearly complete pinniped skeleton from the middle Miocene Okoppezawa Formation (*ca* 16.3–13.9 Ma), Hokkaido, northern Japan, is described as the holotype of *Allodesmus uraiporensis* sp. nov. The new species is distinguishable from other species of the genus by having the palatine fissure (incisive foramen) that is located anterior to the canine, an anteriorly located supraorbital process of the frontal, and by having the calcaneum with a developed peroneal tubercle. Our phylogenetic analysis suggests that the subfamily Allodesminae are represented by two genera, *Atopotarus* and *Allodesmus*, and the latter genus is represented by at least six species; *Al*. *kernensis*, *Al*. *sinanoensis*, *Al*. *naorai*, *Al*. *packardi, Al. demerei* and *Al. uraiporensis* sp. nov. *Allodesmus uraiporensis* sp. nov. is one of the oldest and the northernmost record of the genus in the western North Pacific, and it suggests that the diversification of the genus in the western North Pacific was synchronous to the time of their diversification in the eastern North Pacific.

## 1. Introduction

*Allodesmus* (Desmatophocidae: Allodesminae) is an extinct genus of pinnipeds (Mammalia: Carnivora) known from the middle to late Miocene of the North Pacific (e.g. [1–14]). *Allodesmus* is generally considered to belong to the extinct family Desmatophocidae (e.g. [13–17]), but the interspecific relationships within the genus has been debated. For instance, Barnes & Hirota [12] described two new genera and three new species within the subfamily Allodesminae, and they recognized four genera and at least eight species within the subfamily in total. By contrast, Kohno [13] recognized *Allodesmus* as the only genus of the subfamily, and accepted only five species within the genus (note that the study of Kohno [13] was carried out almost in parallel that of with Barnes & Hirota [12], and, therefore, the latter was not included in the former). Later, Kohno *et al*. [18] suggested that the genus-level classification of the Allodesminae should be re-considered carefully due to ontogenetic changes and sexual dimorphism. Regarding the phylogenetic relationships among the Allodesminae, Kohno [13] performed the first detailed phylogenetic analysis for the five species of *Allodemus* (*Al. courseni*, *Al. kernensis*, *Al. sinanoensis*, *Al. packardi*, *Al. naorai*) with other pinniped taxa based on cranial characters, and found that *Al. kernensis* and *Al. sinanoensis* form the ‘long head' subgroup, whereas *Al. naorai* and *Al. packardi* form the ‘broad head' subgroup. These two groups were sister groups, and *Al. courseni* appeared as a separate basal species within the genus. After Barnes & Hirota [12] and Kohno [13], studies of allodemines ceased for almost 20 years (except [15] for the Desmatophocinae), and accumulation of information of these enigmatic pinnipeds stagnated.

Recently, Boessenecker & Churchill [14] described a new species *Al. demerei* and revised the taxonomy of the Desmatophocidae. They recognized two genera (i.e. *Atopotarus* and *Allodesmus*) within Allodesminae and six *Allodesmus* species (i.e. *Al. kernensis*, *Al. sinanoensis*, *Al. sadoensis*, *Al. naorai*, *Al. packardi* and *Al. demerei*) based on their phylogenetic analysis and morphological comparisons with considerations on ontogenetic changes and sexual dimorphism.

The fossil records of Allodesminae are limited to Japan and the west coast of North America since Kellogg [1] described the first specimen of this taxon in 1922 [12,13,19], and most of them are not sufficiently understood partly due to the poor preservation of the postcranial material. Among the species of Allodesminae, only three well-preserved skeletons are known from California and Washington states. The first well-preserved specimen is an anterior half skeleton that was initially described as the holotype of *Atopotarus courseni* by Downs [20]. Mitchell [7] regarded this genus as a junior synonym of *Allodesmus*, but later works have recognized it as a distinct genus. The second well-preserved specimen is the holotype of ‘*Al. kelloggi*' (a junior synonym of *Al. kernensis*) described by Mitchell [7] which is an articulated partial skeleton. The third is an anterior half skeleton of the holotype of *Al. demerei* by Boessenecker & Churchill [14]. However, other species are known only from cranial material.

**Figure 1.**
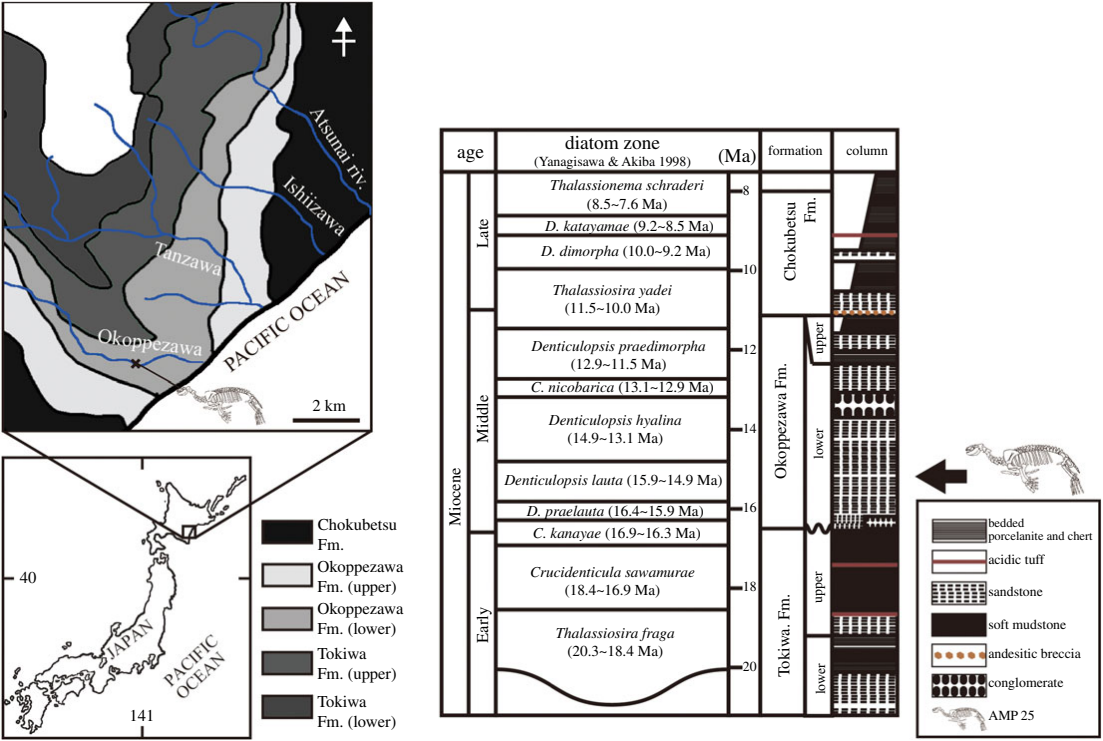
Locality map and stratigraphy of the holotype (AMP 25) of *Al. uraiporensis*, based on Tanaka *et al*. [22].

In 1991, a well-preserved pinniped skeleton belonging to *Allodesmus* was collected from the middle Miocene Atsunai Formation in the southeast Atsunai, Urahoro Town, Tokachi County, Hokkaido, northern Japan (figure 1) [21]. Later, Tanaka *et al*. [22] studied its taphonomy and pointed out that the specimen represents the northernmost occurrence of *Allodesmus* in Japan and possibly the oldest occurrence of the genus in the western North Pacific. Otsuka & Nakaya [23] interpreted this individual as a male based on the morphology of the pelvis. However, because of incomplete preparation of the specimen and scarcity of comparative skeletons of the genus as mentioned above, the specimen remained undescribed for a long time since its discovery.

Because the specimen from the Atsunai Formation is the third example of a nearly complete skeleton of *Allodesmus*, it has a potential to contribute to our further understanding of the evolution and diversification of the genus. Accordingly, we describe this specimen now stored at the Ashoro Museum of Paleontology where it bears the registration number AMP 25, and analyse its phylogenetic relationships among the pinnipeds. This published study and the nomenclatural acts it contains have been registered in Zoobank. The LSID for this publication is: urn:lsid:zoobank.org:pub:EFF71195-756A-4581-B73F-D5773622CF74.

## 2. Material and methods

The following specimens of the genus *Allodesmus* were examined personally for the comparison: LACM 4320 (a referred skeleton of *Al. kernensis*); NMJH A-636-1-1-16 (formerly N-001, holotype cranium of *Al. naorai*); MSFM 00006 and MSFM 00002 (holotype and referred crania of *Al. sinanoensis*); LACM 33561, a cast of CAS 4371A (holotype cranium of *Al. packardi*); NMNS-PV 21915, a cast of SICC 0001 (holotype cranium and mandible of *Al. sadoensis*); and LACM 1376 (holotype skeleton of *Atopotarus courseni*). Definitions of measured portions followed Kellogg [2], Von den Driesch [24] and Kohno [13], and the measurements were taken using a digital caliper and are listed in electronic supplementary material, tables S1–S21.

For the phylogenetic analysis, morphological (osteological) characters and character states were taken from Berta & Wyss [25], Kohno [13] and Tanaka & Kohno [26], and four characters were added as new characters. In total, 100 characters for 15 ingroup and five outgroup taxa were analysed in this study (see electronic supplementary material). A complete list of analysed taxa/specimens is presented in electronic supplementary material. Characters and tree data were prepared on Mesquite 3.04 [27], and cladistic analyses were performed on PAUP* 4.0a152 [28]. All characters were unweighted and unordered. A heuristic search with 10 000 replicates was conducted, using tree bisection reconnection (TBR). Character distributions were determined using the DELTRAN optimization of PAUP. Bootstrap support value was calculated by using symmetrical resampling with 1000 replicates.

Institutional abbreviations:

AMP: Ashoro Museum of Paleontology, Hokkaido, Japan.
CAS: California Academy of Sciences, California, USA.
LACM: Natural History Museum of Los Angeles County, Los Angeles, California, USA.
MSFM: Matsumoto Shiga Fossil Museum, Nagano, Japan.
NMJH: National Museum of Japanese History, Chiba, Japan.
NMNS: National Museum of Nature and Science, Ibaraki, Japan.
SICC: Sado Island Community Center, Sado, Niigata, Japan.
USNM: National Museum of Natural History, Smithsonian Institution, Washington, DC, USA.

## 3. Systematic palaeontology

Mammalia Linnaeus, 1758

Carnivora Bowdich 1821

Pinnipedia Illiger, 1811

Family Desmatophocidae Hay, 1930

Subfamily Allodesminae (Kellogg, 1931)

Genus *Allodesmus* Kellogg, 1922

*Allodesmus* Kellogg, 1922, p. 26

*Atopotarus* (part). Packard, 1962, p. 30

*Brachyallodesmus* (part). Barnes and Hirota, 1995, p. 332

*Megagomphos* (part). Hirota and Barnes in Barnes and Hirota, 1995, p. 335

**Emended diagnosis of genus.** Presence of prenarial shelf; presence of facet for tympanohyal within hyoid fossa; transversely expanded posterior lacerate foramen; weak and smooth lingual cingulum of P1–2; single-rooted P3–4 and M1; medially prominent calcaneal tuber, overlapping entocuneiform/mesocuneiform articulation. Dental formula: I3 · C1 · P4 · M1–2/I2 · C1 · P4 · M1–2 [13,14].

**Type species.** *Allodesmus kernensis* Kellogg, 1922

**Included species.** *Allodesmus kernensis* Kellogg, 1922 (incl. *Al. kelloggi* Mitchell, 1966 and *Al*. *gracilis* Barnes in Barnes and Hirota, 1995, see Deméré & Berta [16]), early middle Miocene, California; *Al. sinanoensis* (Nagao, 1941) Mitchell, 1968 (incl. *Al*. *sadoensis* Hirota in Barnes and Hirota, 1995 and *Al. megallos* Hirota in Barnes and Hirota, 1995, as will be discussed below), late middle Miocene, Japan; *Al. packardi* Barnes, 1972, middle Miocene, California; *A. naorai* Kohno, 1996, late middle Miocene, Japan; *Al. demerei* Boessenecker and Churchill, 2018, late Miocene, Washington; and *Allodesmus uraiporensis* sp. nov., early to middle Miocene, Japan.

*Allodesmus uraiporensis* sp. nov.

*Allodesmus* sp. Tanaka *et al*., 2004; Tanaka, 2008

*LSID*. urn:lsid:zoobank.org:pub:EFF71195-756A-4581-B73F-D5773622CF74

**Diagnosis of species.** *Allodesmus uraiporensis* sp. nov. is distinguished from all other *Allodesmus* by having the palatine fissure (incisive foramen) that is located anterior to the canine (character 7 : 1) and an anteriorly located supraorbital process of the frontal (character 20 : 0), a developed peroneal tubercle of the calcaneum (character 98 : 1); from *Al. kernensis*, *Al. sinanoensis Al. demerei* and *Al. naorai* by having rounded infraorbital foramen (character 9 : 0); from *Al. naorai* by having M^2^ that is smaller than premolars (character 86 : 0); from *Al. kernensis*, *Al. sinanoensis* and *Al. demerei* by having a developed but not laterally exaggerated prenarial shelf (character 1 : 1) and a flat palate (character 12 : 0), posteriorly very wide palatal margins of the maxilla (character 13 : 2); from *Al. packard* by having the smaller size of M^2^ than premolars (character 86 : 0) and the small supraorbital process of the frontal (character 19 : 0).

**Figure 2.**
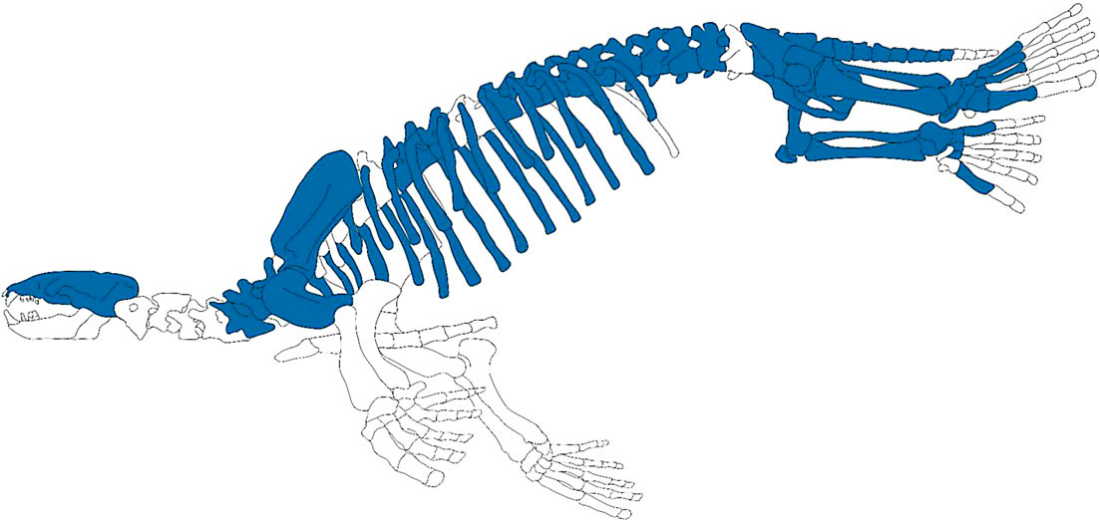
Reconstruction of *Allodesmus* skeleton. Blue parts indicate preserved bones of the holotype (AMP 25) of *Allodesmus uraiporensis*.

**Holotype.** AMP 25: partial skeleton of an adult male individual [22,23]; left half of the cranium, cervical vertebrae 5–7, thoracic vertebrae 6–8 and 11–15, lumbar vertebrae 1–4, sacrum, caudal vertebrae 1–8 and two of uncertain positions, three sternebrae (uncertain position), left ribs 1 and 4–15, right ribs 1–14, left scapula, left humerus, middle phalanx of manus (uncertain position), pelves, femora, patellae, tibiae, fibulae, astragali, calcanei, right cuboid, naviculars, entocuneiforms, mesocuneiforms, ectocuneiforms, left metatarsals 1 and 5, right metatarsals 1 and 3–5, a proximal phalanx of pes (uncertain position), two middle phalanges of pes (uncertain positions) (figure 2).

**Type Locality.** An outcrop at Okoppezawa River about 2 km from the mouth of the river, Atsunai, Urahoro Town, Tokachi, Hokkaido, northern Japan, 44°49′30′′ N, 143°46′30′′ (figure 1) [21,22,29].

**Horizon and age.** The holotype came from the middle Miocene Okoppezawa Formation of the Atsunai Group [22,29]. According to Tada & Iijima [30], the Atsunai Group is subdivided into the Tokiwa, Okoppezawa, Chokubetsu, Atsunai and Shiranuka Formations in ascending order. The geologic age of the Atsunai Group spans from the early Miocene to late Pliocene based on the diatom biostratigraphy; i.e. *Thalassiosira fraga* zone∼*Neodenticula koizumii* zone [22] (figure 1). The Okoppezawa Formation is subdivided into the lower Ishiizawa Sandstone Member and the ‘Upper Sand/Mud alternating Member’ [30], and the holotype of *Allodesmus uraiporensis* sp. nov. was found *in situ* in the former [22]. The Ishiizawa Sandstone Member coarsens upward from the sandy silt to the coarse-to-medium sand; the lowermost layer includes glauconic sandstone, whereas the middle part includes several shell beds representing the Atsunai Fauna, which includes shallow marine taxa [30]. Tanaka *et al*. [22] considered the geological age of the lower part of the Ishiizawa Sandstone Member where the holotype was obtained to be 16.3–13.5 Ma (the early middle Miocene) based on diatoms (*Denticulopsis praelauta*∼*D. hyalina*) [31] and nannofossils (N8–N13) [32] (figure 1).

**Etymology.** The species name is derived from ‘Uraiporo’, an original place name for the current town of Urahoro given by the Japanese native tribe Ainu who live mainly in Hokkaido. ‘Uraiporo' in Ainu refers to a big fish ground where fish are always gathering, plus Latin suffix for place; -ensis.

## 4. Description

**Figure 3.**
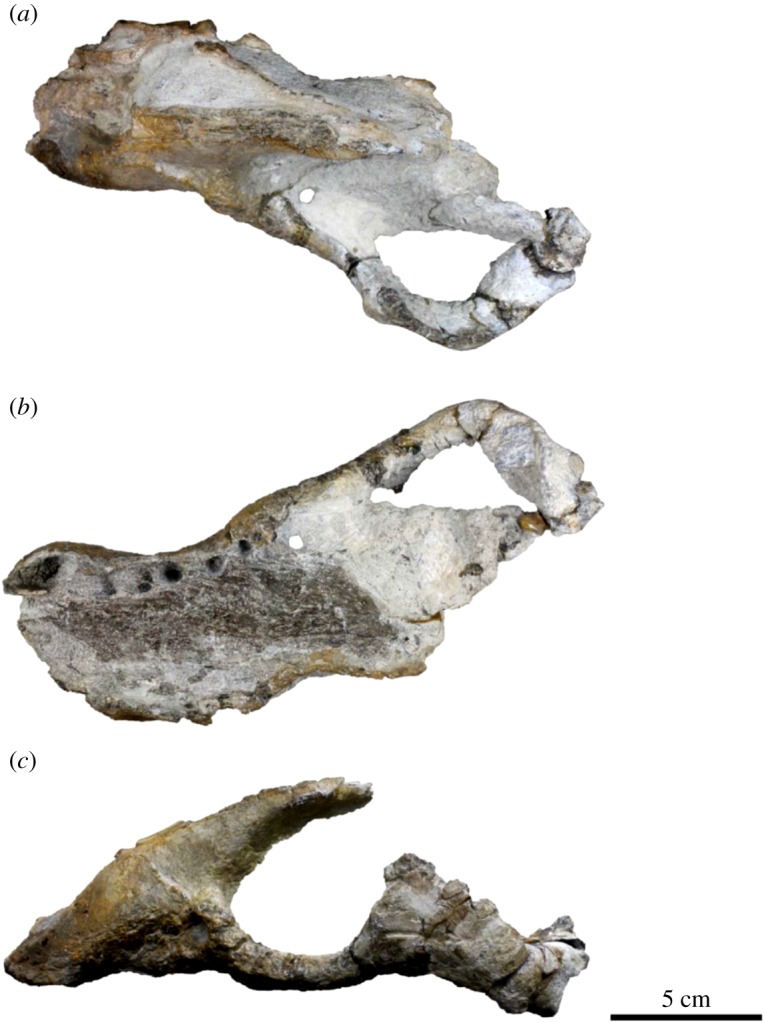
Skull of the holotype (AMP 25) of *Allodesmus uraiporensis*. (*a*) dorsal, (*b*) ventral and (*c*) lateral views.

**Figure 4.**
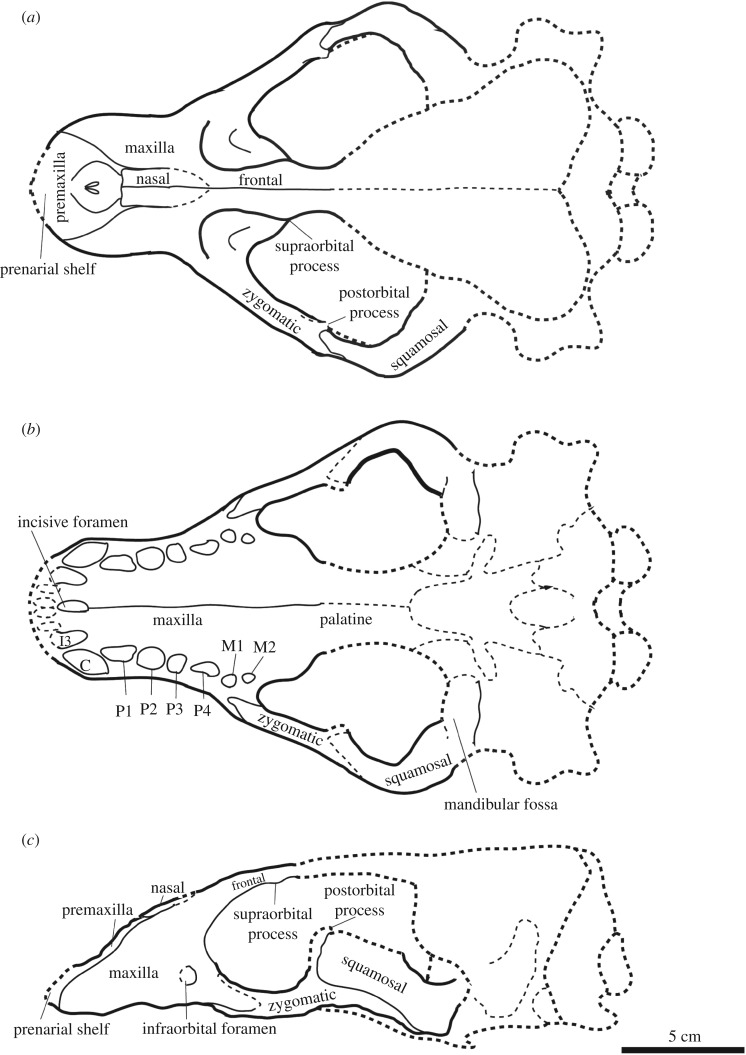
Reconstruction of skull of the holotype (AMP 25) of *Allodesmus uraiporensis*. (*a*) dorsal, (*b*) ventral and (*c*) lateral views. Solid lines indicate those elements that are preserved on at least one side of the holotype, and other missing parts are represented by dashed lines.

Cranium (figures 3*a*–*c* and 4*a*–*c*; electronic supplementary material, table S1): the left side of the snout and left zygomatic arch, as well as the palate, are well preserved. Cranial sutures are mostly fused and hardly traced. In addition to the suture closure of the cranium, the upper canine is large relative to the cheek tooth row, suggesting that the specimen belongs to an adult male [33].

The premaxilla forms the large narial opening and has thick margins in anterior view and is retracted posteriorly along its ascending process. The posterior end of the narial opening is located above P3. The dorsal part of the premaxilla anterior to the narial opening forms a prenarial shelf. The prenarial shelf is developed but not laterally exaggerated. The length of the shelf (posterior end of the narial opening to the anterior end of the skull) is approximately 42% of the pre-zygomatic skull length (anterior end of the zygomatic arch to the anterior end of the skull), and the maximum width of the prenarial shelf (distance from the labial rim of the C1 alveolus to the midline) is 77% of the skull width (anterior end of the zygomatic arch to the anterior end of the skull). The ascending process extends posterodorsally and overlaps the lateral edge of the nasal for a short extent. The incisive foramen is a single opening and located anterior to the canine. Its posterior margin is almost aligned with that of the alveoli of I3.

The maxilla forms the lateral wall of the rostrum, a palatal portion along the cheek tooth row and a posteroventral portion of the orbital rim. The antorbital process is absent. The infraorbital foramen is relatively enlarged and rounded in outline, and it is located above P4. There is no indication of the nasolabial fossa on the cheek region. The ventral floor of the infraorbital foramen extends posteriorly into the orbital rim. The palatal region of the maxilla is very wide and flat. The palatal margin is posteriorly very wide. The median palatine suture of the maxilla is clear and it extends to the anterior portion of the palatine.

The palatine is relatively wide and expands posterolaterally; the width between the medial edges of C1 alveoli is only 49% of that between the medial edges of M1 alveoli. The suture between the palatine is still recognizable but the suture between the palatine and the maxilla is almost fused and uncertain. The posterior margin of the palatine is missing.

The jugal is relatively straight and widens posteriorly with the maximum width at the postorbital process. The postorbital process is dorsally projecting. The anteroventral process is elongate and reaches to the alveolus of M1.

The squamosal is mostly missing except for its zygomatic process. The zygomatic process of the squamosal is dorsoventrally thick, and its anterior tip is square and interlocking with the postorbital and posteroventral processes of the jugal.

The nasal is dorsally flat and broad. Its anterior edge is broken. The surface of its posterior end is damaged, but it penetrates to the mid-portion of the frontal.

The frontal is badly broken, and only its anterior-most portion is preserved. It is narrow, and the supraorbital process is a small bump and anteriorly located.

Dentition (figures 3*a*–*c* and 4*a*–*c*; electronic supplementary material, table S1): no tooth is preserved. Remaining alveoli on the left side of the cranium are well preserved except for those of incisors. The alveoli indicate that all the teeth are single rooted. The poorly preserved alveolus for I3 is strongly procumbent and elliptical in outline. The alveolus for C1 is relatively large and procumbent. It projects labially and is located outside the cheek tooth row. The alveolus for P1 is also strongly procumbent and elliptical in outline. There is a narrow diastema between C1 and P1. The alveolus for P2 is also procumbent. The alveolus for P3 is slightly procumbent and slightly smaller than P2. The alveolus for P4 is elliptical in outline. The alveoli for the molars are distinctively smaller and shallower than those of the premolars. The outer edge of the alveolus for M1 projects slightly more labial than the row of those of premolars. The alveolus for M2 is the smallest and shallowest of the cheek teeth.

### 4.1. Axial skeleton

**Figure 5.**
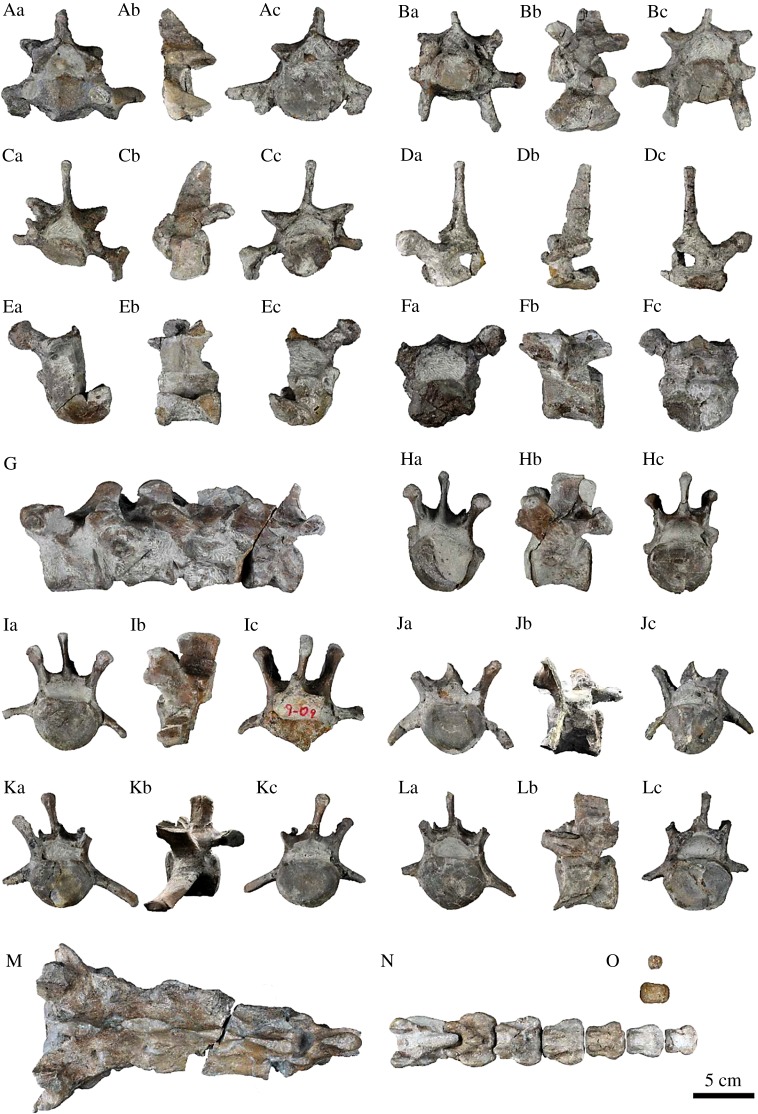
Axial skeleton of the holotype (AMP 25) of *Allodesmus uraiporensis*. From left to right, anterior, lateral and posterior views. (A–C) cervical vertebrae. (Aa–Ac) fifth; (Ba–Bc) sixth; (Ca–Cc) seventh; (D–H) thoracic vertebrae; (Da–Dc) sixth; (Ea–Ec) seventh; (Fa–Fc) eighth; (G) 11th to 14th in lateral view; (Ha–Hc) 15th. (I–L) lumber vertebrae. (Ia–Ic) first; (Ja–Jc) second; (Ka–Kc) third; (La–Lc) fourth. (M) Sacrum in dorsal view. (N) First to eighth caudal vertebra in dorsal view. (O) Two caudal vertebrae of uncertain positions.

Cervicals (figure 5A*a*–Cc; electronic supplementary material, table S2): C5, C6 and C7 were preserved, of which C5 is the smallest and C6 the largest among them. C5 is incomplete, and the anterior half of the centrum and the ventral tubercle are broken. In posterior view, the centrum of C5 is subcircular in outline. The neural spine is low and slightly inclined anteriorly. The prezygapophysis is broken. The postzygapophysis is short, and its facet is small and subcircular in outline. The transverse foramen is ovoid for C5 as well as C6. C6 is well preserved, and the anterior surface of the centrum is smaller than the posterior one. There is an anteroposteriorly extended narrow ridge on the midline of the ventral surface of the centrum. The neural spine is broken. The lateral edge of the prezygapophysis is prominent. The postzygapophysis is larger than that of C5, and projects posteriorly. The dorsal tubercle of the transverse process is knob-like, and it projects ventrolaterally and posteriorly. The ventral tubercle is an anteroposteriorly expanded, fan-shaped, thick plate. C7 is incomplete, and its anterodorsal part of the centrum is broken. The centrum is smaller than those of C5 and C6, and it is more circular in outline. The neural spine is relatively high, and it projects dorsally. The prezygapophysis is broken. The postzygapophysis is expanded, and it projects posterolaterally. Its facet is large and subcircular in outline. The transverse process of C7 is long and projects laterally, and its distal end is expanded dorsoventrally.

Thoracics (figure 5Da–Hc; electronic supplementary material, table S3): originally 10 thoracic vertebrae from T4 to T8 and T12 to T15 had been preserved on AMP 25, but T4 and T5 (illustrated in [28]) are now missing and presumed lost. T6 is incomplete, and only the neural spine, neural arch and transverse process are preserved. The neural spine is nearly upright and high. The neural arch is notched posteriorly. The transverse process is short and knob-like. T7 is also incomplete, and much of the right half is broken and missing. The centrum is laterally wide and subcircular in anterior and posterior views. The anterior surface bears a small depression in the centre, and it is smaller than that on the posterior surface. The neural spine, the prezygapophysis and the postzygapophysis are broken. The transverse process is morphologically similar to that of T6. The anterior costal facet is dorsoventrally long and subcircular in outline, whereas the posterior costal facet is a rectangle with rounded corners. T8 is relatively well preserved, and it is morphologically similar to T7, but the centrum is larger. The neural spine, the prezygapophysis and the postzygapophysis are broken. The transverse process projects more horizontally. The costal facets are similar to those of T7 in morphology. T11 to 14 are articulated in the block. Those centra are similar in size and in morphology to each other. The neural spines are low in T11 and T12. Those of T13 and T14 are broken. The transverse process of T11 is well developed, but it is reduced in subsequent thoracics; instead, the mammillary process is pronounced in T12–15. The facet of the pre- and postzygapophysis is subcircular and faces laterally on T11–14. The costal facet is circular on T11–13, but it is subcircular on T14. T15 is nearly complete. It is similar to T14 in morphology. The neural spine is higher than that of T11. The mammillary process is more developed than that of T14. The costal facet is reduced to a small tubercle.

Lumbars (figure 5Ia–Lc; electronic supplementary material, table S4): L1 to L4 are preserved. L1 articulates to the last thoracic, but, L4 does not articulate to the sacrum, indicating that L4 is not the last lumbar and L5 was originally present. The preserved four lumbars are very similar to each other and vary only in minor details. The centrum of each lumbar is subcircular in outline, but its lateral margins are slightly depressed internally. The neural spine is a square plate, and its dorsal edge is transversely thickened. The neural canal is large and semicircular. The mammillary process is well developed. The prezygapophyses are widely separated and larger than those of thoracics. The facet of the prezygapophysis is elongate and subcircular in outline. The facet of the postzygapophysis is also elongated and subcircular in outline. The transverse process projects laterally, and that of L1 is the shortest among the lumbars. It is longer on the posterior lumbars and curved anteroventrally in L1 and L2. That of L3 and L4 is straight.

Sacrum (figure 5M; electronic supplementary material, table S5): the sacrum is well preserved. In dorsal view, the sacrum is a large isosceles triangle that narrows caudally. The sacral ala of the first one is laterally wide. The anterior margin of the lateral edge is laterally projected. The auricular surface is long anteroposteriorly, and it reaches to the sacral ala of the second one. The sacral canal is triangle with rounded corners in anterior view. The superior articular facet is square with rounded corners in outline, and the posterior edge projects caudally. The spinous processes are connected to each other and create a plate-like crest. The first sacral process is the highest. The processes become gradually lower and longer posteriorly; those of the third and fourth ones are at about the same height. The four processes are fused, but the median and medial sacral crests are less developed. Dorsal sacral foramina are small and rounded, whereas ventral sacral foramina are larger than the dorsal ones.

Caudals (figure 5N,O; electronic supplementary material, table S6): caudal vertebrae 1–8 and two caudal vertebrae of uncertain position are preserved. The first caudal vertebra does not articulate with the fourth sacral vertebra but obliquely fuses with it, so this condition is probably pathological. The centrum of the first sacral vertebra is subcircular in outline, but those of the rest of the sacral vertebrae are more circular. On the first, second and third caudal vertebrae, the neural spine is low and laterally thick, and it is notably thick in dorsal view. The centrum and neural spine become gradually smaller posteriorly, and the fourth caudal vertebra is the last vertebra with neural spine. The pre- and postzygapophysis of the first caudal vertebra are well developed, and the postzygapophysis projects caudally. The prezygapophysis of caudal vertebrae 1–7 become gradually smaller caudally, and they are absent in the caudals posterior to the eighth one. The postzygapophysis is also absent in the caudals posterior to the third one. The transverse process forms a lateral ridge. The caudals posterior to the eighth one are pisiform.

**Figure 6.**
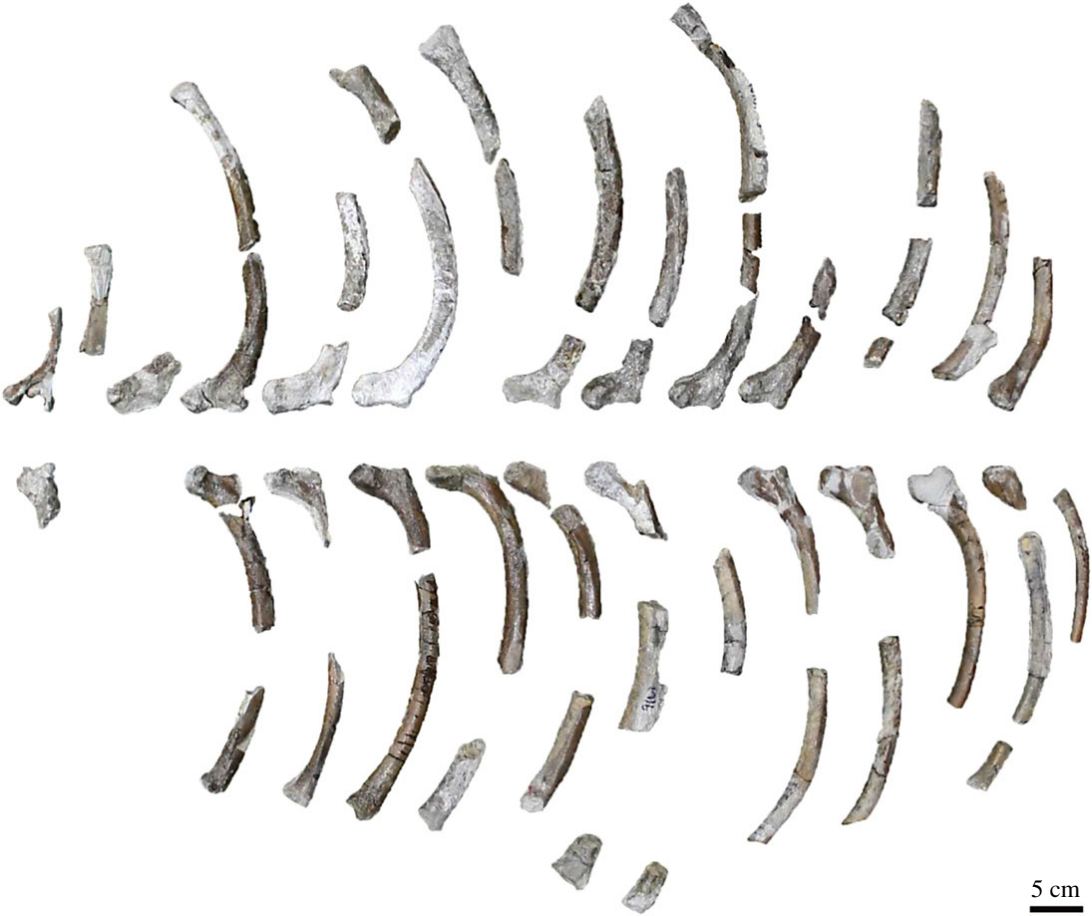
Ribs of the holotype (AMP 25) of *Allodesmus uraiporensis* in anterior views.

Ribs (figure 6): all the ribs are damaged, but left ribs 1 and 4–15, and right ribs 1–14 are preserved. The anterior ribs have a wide neck, large capitula and tubercula. The neck becomes narrow posteriorly. The capitula and tubercula are small. The shaft is gently curved. First, second and third ribs have a relatively flattened shaft. Other than these, the shaft has a triangle with rounded corners in cross section. Ribs 14 and 15 have no capitula, and the tubercles of those are knob-like.

**Figure 7.**
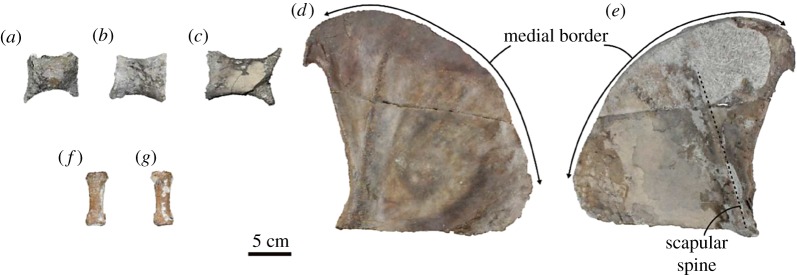
Thorax and forelimb bones of the holotype (AMP 25) of *Al. uraiporensis*. (*a*–*c*) Sternebrae of uncertain positions in dorsal views. (*d*) and (*e*) Left scapula in medial and lateral views. (*f*) and (*g*) Middle phalanx of manus of uncertain position in dorsal and ventral views.

Sterna (figure 7*a–c*): three sternebrae of uncertain position are preserved, but these are badly damaged. The cross section of all preserved sternebrae is subcircular in outline. St-A is about the same size of St-B. Although the anterior and posterior ends of the St-A are laterally flared, those of St-B are not so wide as those of St-A. St-C is the longest among the three sternebrae. Its ends also are laterally flared.

### 4.2. Appendicular skeleton

Scapula (figure 7*d*,*e*): only the left scapula is incompletely preserved. It is thin and fan-shaped. The scapular spine is well developed with a small ventral tilt. The supraspinous fossa is more than twice as wide as the infraspinous fossa. The supraspinous fossa is very smooth, and lacks the spina scapulae accessoria as in Otariidae. The posterior border is thick, and the inferior angle projects posteriorly. The medial border is also thick in its posterior parts, but the edge becomes thinner anteriorly. The subscapular fossa is smooth but uneven. There is an indistinct ridge medial to the posterior border, and the space between the posterior border and the ridge makes a fan-shaped groove. Anterior portion of the subscapular fossa is smoothly concave, but it swells near the medial border.

**Figure 8.**
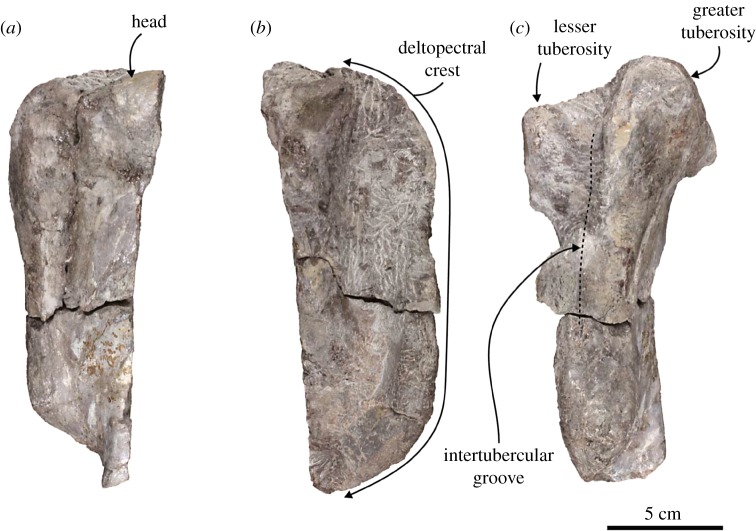
Left humerus of the holotype (AMP 25) of *Allodesmus uraiporensis*. (*a*–*c*) Humerus in lateral, medial and anterior views.

Humerus (figure 8): the proximal portion of the left humerus is preserved. The diaphysis is relatively slender. The greater tuberosity is well developed, but it is slightly lower than the head. It continues to the deltopectral crest, which is anteriorly high and relatively long. The intertubercular groove is deep proximally and gradually shallows distally. The lesser tuberosity is also well developed, but it is slightly lower than the greater tuberosity. The head is mostly broken, but its anterolateral part is convex laterally.

Phalanx of manus (figure 7*f*,*g*; electronic supplementary material, table S7): a middle phalanx of uncertain position is completely preserved. It is short and flat in cross section. Its proximal facet is laterally wide, and the distal surface is semicircular in outline.

**Figure 9.**
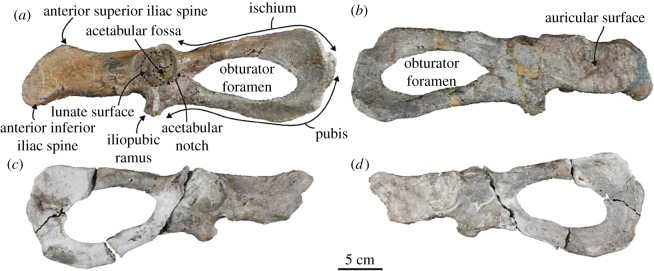
Both pelves of the holotype (AMP 25) of *Allodesmus uraiporensis*. (*a*,*b*) Left pelvis in lateral and medial views. (*c*,*d*) Right pelvis in lateral and medial views.

Pelvis (figure 9; electronic supplementary material, table S8): the left pelvis is completely preserved. It is anteroposteriorly long. The obturator foramen is subcircular in outline. The acetabulum is hemispherical and accommodates a relatively deep acetabular notch at its posteroventral edge. The acetabular fossa is small. The lunate surface is broad. The acetabular border is well developed as a distinctive ridge.

The ilium is shorter than the pubis and ischium, and curves laterally. There are two tubercles on the lateral surface at the posteroventral portion of the ilium. The anterior inferior iliac spine is thick and projects anteroventrally, the posterior superior iliac spine is less developed. The anterior superior iliac spine projects anterodorsally, but it is also less developed. The gluteal surface is gently convex longitudinally and above the two tubercles. The auricular surface consists of three shallow depressions.

The ischium is long anteroposteriorly, and the body is triangular in cross section. It has a less developed ischial spine on the dorsomedial edge. The inferior ramus is plate-like and expanded dorsoventrally. The tuberosity is not distinct.

The pubis is also long anteroposteriorly, and it gently slopes posteroventrally. The iliopubic ramus projects anteroventrally. The portion between the acetabular notch and the anterior edge of the obturator foramen is broad. The body is semicircular in cross section. The inferior ramus is plate-like and expanded dorsoventrally and posteriorly. The symphysial surface is laterally narrow and located at the posteroventral edge. The medial surface of the pubis is nearly flat.

**Figure 10.**
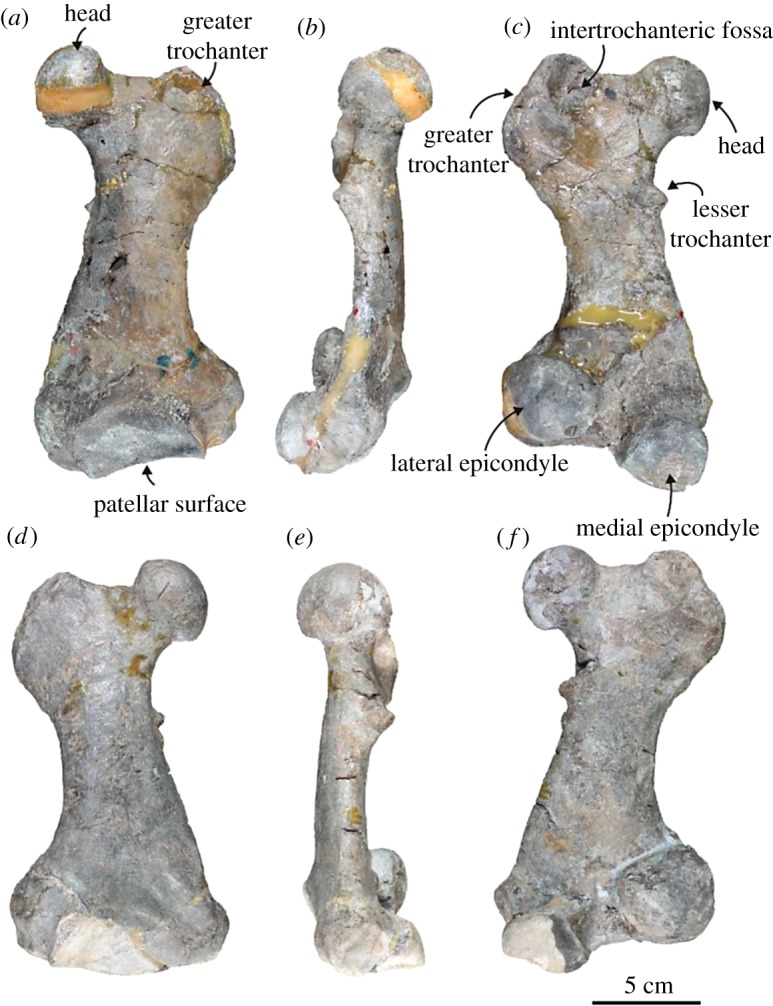
Both femora of the holotype (AMP 25) of *Allodesmus uraiporensis*. (*a*–*c*) Left femur in anterior, medial and posterior views.(*d*–*f*) Right femur in anterior, medial and posterior views.

Femur (figure 10; electronic supplementary material, table S9): both left and right femora are well preserved. The head of the femur is well developed and spherical. The fovea capitis for the ligamentum teres femoris is absent on the head. The neck is short and wide. The greater trochanter is also well developed proximally and laterally. The proximal ends of the greater trochanter and the head are at the same height. The intertrochanteric fossa is shallow. The lesser trochanter is small and triangular, and it projects posteromedially. The shaft is slender and flat anteroposteriorly. The medial and lateral condyles are well developed, and the former is smaller than the latter. The articular facet of the medial condyle is trapezoidal in outline, whereas that of the lateral condyle is circular. The articular facet of the medial condyle continues to the patellar surface, but it is not the case for the lateral condyle. The intercondylar fossa is deep. The medial and the lateral epicondyles are only slightly developed, but the lateral epicondyle is more pronounced than the medial one. The patellar surface is laterally wide and rectangular in outline.

**Figure 11.**
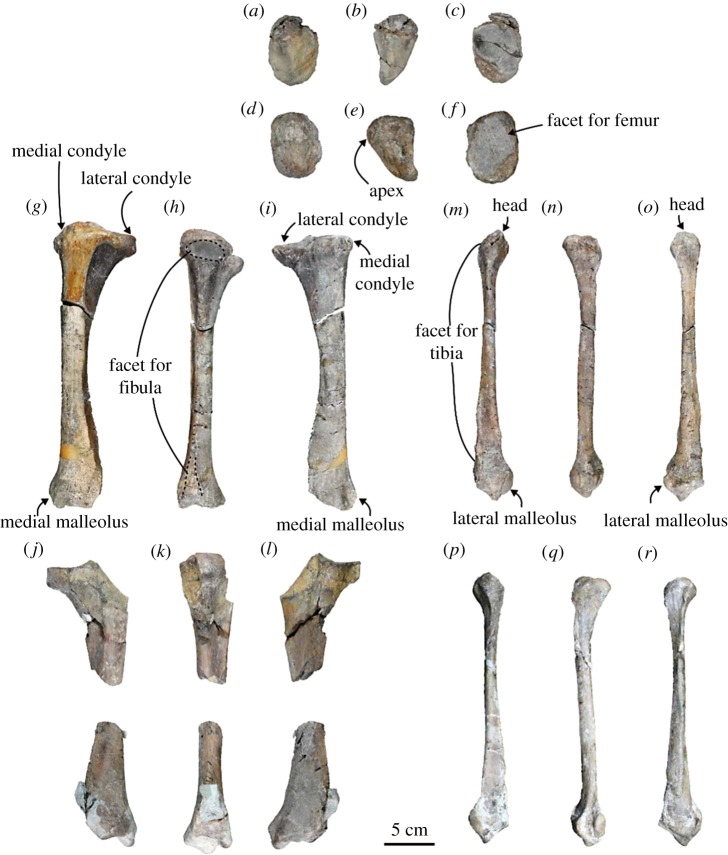
Hindlimbs of the holotype (AMP 25) of *Allodesmus uraiporensis*. (*a*–*c*) Left patella in anterior, medial and posterior views. (*d*–*f*) Right patella in anterior, medial and posterior views. (*g*–*i*) Left tibia in anterior, lateral and posterior views. (*j*–*l*) Right tibia in anterior, lateral and posterior views. (*m*–*o*) Left fibula in anterior, lateral and posterior views. (*p*–*r*) Right fibula in anterior, lateral and posterior views.

Patella (figure 11*a*–*f*; electronic supplementary material, table S10): the right patella is completely preserved. The patella is a large, subcircular bone, and dorsoventrally long rather than laterally wide. The apex of the patella is well developed and pointed proximally. The anterior surface is rugose. The facet is dorsoventrally concave, and it is subcircular with an oblique axis running from the dorsomedial to ventrolateral corners.

Tibia (figure 11*g*–*l*; electronic supplementary material, table S11): the left tibia is well preserved. It is unfused to the fibula at its proximal end. The shaft of the tibia is nearly straight, but the fibular side of the shaft is more concave than the medial side. The proximal and distal ends of the shaft are expanded. The medial condyle is smaller than the lateral condyle, and its superior facet forms a fan-shaped articular facet. The articular facet of the medial condyle is depressed. On the other hand, the superior articular facet of the lateral condyle is large and anteroposteriorly subcircular. The inferior articular facet is dorsally long and anteroposteriorly subcircular. Both the medial and lateral intercondylar tubercles are not pronounced, but the latter is more developed than the former. The crest of the medial edge is extended along the shaft at the anterior edge of the shaft. There is the tibial tuberosity on the medial edge at the distal end. The anterior tibial fossa is shallow, whereas the posterior tibial fossa is deep. A distal half of the interosseous border forms a sharp crest, and there is a facet for the fibula at the distal end. This facet is rough and tall isosceles triangular with acute proximal angle. In addition, there is a small crescent-shaped articular facet, which continues to the distal facet of the tibia. The distal facet forms a shallowly concave saddle, fitting to the trochlea of the astragalus, and it has a weakly downward process at the anteromedial edge. The medial malleolus is well developed. There is a distinct malleolar groove between the medial malleolus and tibial tuberosity.

Fibula (figure 11*m*–*r*; electronic supplementary material, table S12): both left and right fibulae are cracked on the shaft, but almost completely preserved. It is slender, and has massive ends. In proximal view, the head is triangular, linear at a lateral edge and projects slightly upward. Its apex is less developed. There is a sharp crest at the anterior edge at the proximal portion of the shaft, but it disappears distally. The interosseous border is also crest-like at the proximal portion, and this crest reduces similarly at the middle portion of the shaft, but it is crested again at the distal portion. There is a shallow concavity between the interosseous and the anterior edge. There is a triangular facet for the tibia at the distal end of the interosseous crest. A rectangular facet located below the tuberosity continues to the facet of the malleolus. The lateral surface of the distal end has two well-developed tuberosities, of which the anterior one is larger. There is a deep groove between the two tuberosities. The lateral malleolus projects distally so that the inferior facet is oblique. The inferior facet is trapezoidal, and it extends distally for the articulation with the lateral surface of the astragalar trochlea.

**Figure 12.**
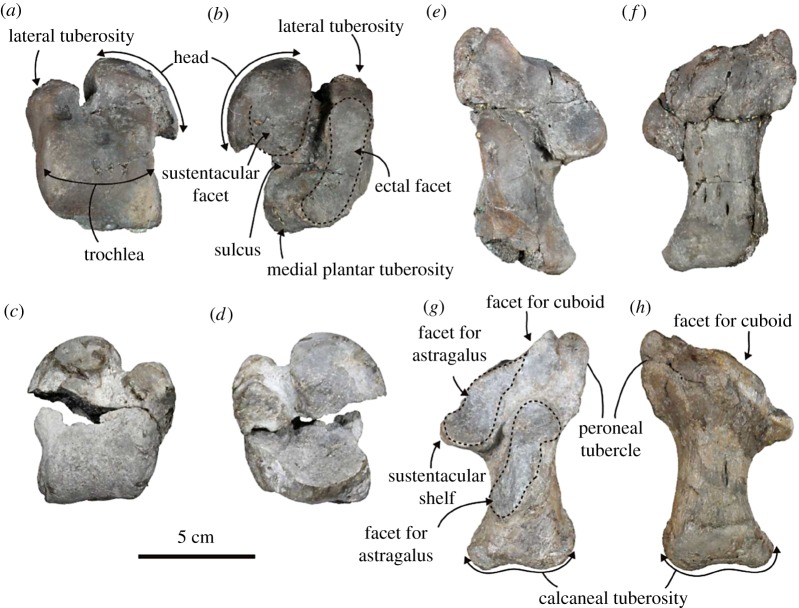
Both astragali and calcanei of the holotype (AMP 25) of *Allodesmus uraiporensis*. (*a*,*b*) Left astragalus in dorsal and plantar views. (*c*,*d*) Right astragalus in dorsal and plantar views. (*e*,*f*) Left calcaneum in dorsal and plantar view. (*g*,*h*) Right calcaneum in dorsal and plantar views.

Astragalus (figure 12*a*–*d*; electronic supplementary material, table S13): the left astragalus is well preserved except for a tip of the lateral tuberosity. The lateral tuberosity is projected anteriorly. The head is large for the size of the body. The neck is short and wide. The facet for the navicular is anteroposteriorly wide and convex anteromedially. It continues to the rounded sustententacular facet. The astragalar sulcus is deep and J-shaped. The ectal facet is an anteroposteriorly elongated crescent in shape, and its posterior end is projected plantarly. The trochlea is large and saddle-shaped. Its lateral part continues to the anteriorly projected lateral tuberosity. A small and horseshoe-shaped depression is located at the centre of the anterior face of the trochlea. The medial plantar tuberosity is projected.

Calcaneum (figure 12*e*–*h*; electronic supplementary material, table S14): the right calcaneum is well preserved. It is relatively slender and elongated anteroposteriorly. The peroneal tubercle is developed. The facet for the cuboid is large and subcircular in outline. It is connected to the anterior facet or the astragalus, which is shaped like a tear-drop. The sustentacular shelf is well developed. The posterior facet for the astragalus is elongated anteroposteriorly, and its posterior part slopes medially. The calcaneal groove is deep at the posterior end. The calcaneal tuberosity is well developed and medially prominent. It has well-developed lateral and medial processes separated by a shallow, short groove on the posterior surface. There is an elongate pit on the medial side of the plantar surface of the body.

**Figure 13.**
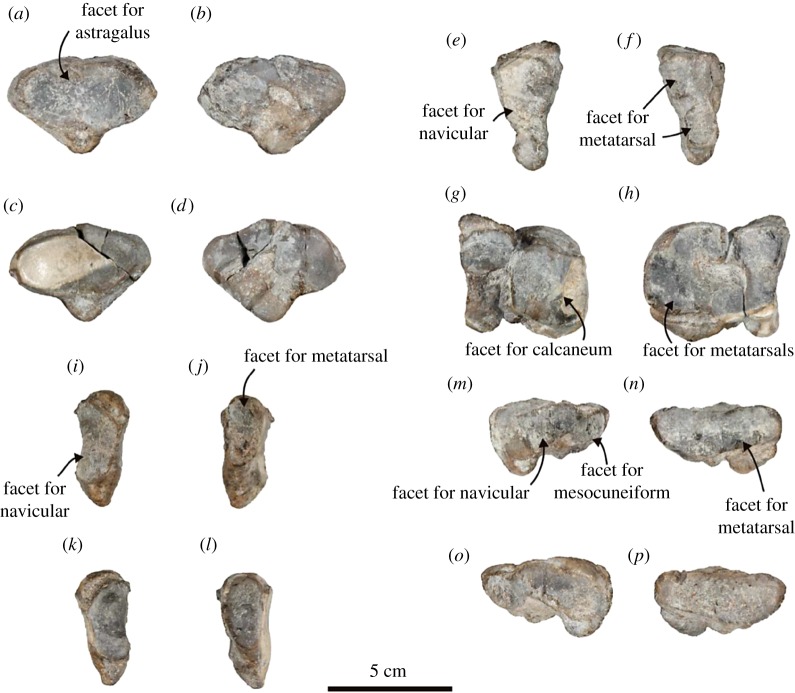
Distal tarsals of the holotype (AMP 25) of *Allodesmus uraiporensis*. (*a*,*b*) Left navicular in posterior and anterior views. (*c*,*d*) Right navicular in posterior and anterior views. (*e*,*f*) Left ectocuneiform in posterior and anterior views. (*g*,*h*) Right ectocuneiform and cuboid in posterior and anterior views. (*i*,*j*) Left mesocuneiform in posterior and anterior views. (*k*,*l*) Right mesocuneiform in posterior and anterior views. (*m*,*n*) Left entocuneiform in posterior and anterior views. (*o*,*p*) Right entocuneiform in posterior and anterior views.

Navicular (figure 13*a*–*d*; electronic supplementary material, table S15): the right navicular is well preserved. It is an inverted, short isosceles triangle with rounded corners in anterior and posterior views. The facet for the astragalus is anteriorly concave and rounded, and it is laterally wide. There is a distinct subcircular pit on the centre of the distal surface. It has a plantar tuberosity, and a small and rounded pit is situated on the proximal surface of that tuberosity.

Cuboid (figure 13*g*,*h*; electronic supplementary material, table S16): the right cuboid is preserved. The cuboid is anteroposteriorly long, and irregular polygonal in lateral view. The facet for the navicular is anteroposteriorly long. The facet for the calcaneum is broad dorsoplantarly. The facet for the metatarsals is C-shaped (the C opens medially) and forms a laterally long and rectangular fossa together with the ectocuneiform. There is a short groove for the tendon of fibularis longus on the plantar surface.

Ectocuneiform (figure 13*e*–*h*; electronic supplementary material, table S17): both left and right ectocuneiforms are completely preserved. It is long and dorsoventrally rectangle in lateral and medial views. The facet for the navicular is long plantarly. The facet for the mesocuneiform is small and elongated plantarly. The facets for the cuboid are separated into four small facets. Each is located at a corner. Two facets at the distal corner and the facet for the metatarsal are overlapped. The facet for the metatarsal is separated upward and downward by a shallow groove. The plantar one is concave and dorsoventrally rectangular, and another one is also concave and laterally rectangular.

Mesocuneiform (figure 13*i*–*l*; electronic supplementary material, table S18): both left and right mesocuneiforms are completely preserved. It is the smallest tarsal bone. The facet for the entocuneiform is elongated anteroposteriorly. The facet for the navicular is rounded and elongated plantarly. The facet for the ectocuneiform is a tiny facet, and it is quadrilateral in outline. The facets for the navicular and ectocuneiform are overlapped as in Odobenidae. The facet for the metatarsal is small and elongated plantarly.

Entocuneiform (figure 13*m*–*p*; electronic supplementary material, table S19): both left and right entocuneiforms are completely preserved. The entocuneiform is fan-shaped in dorsal and plantar views. The facet for the navicular and for the mesocuneiform is overlapped. The facet for the navicular is wide. The facet for the mesocuneiform is smaller than that for the navicular. The facet for the first metatarsal is laterally elongated.

**Figure 14.**
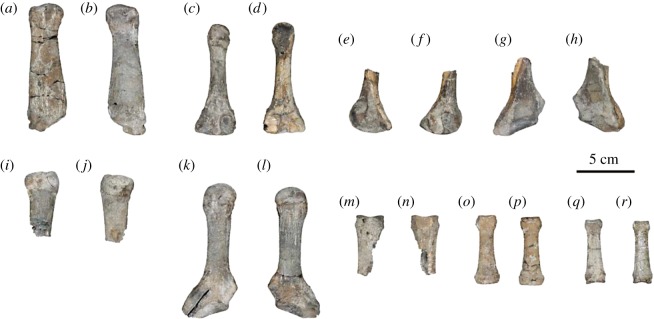
Metatarsals and phalanges of the holotype (AMP 25) of *Allodesmus uraiporensis*. (*a*,*b*) Right first metatarsal in dorsal and ventral views. (*c*,*d*) Right third metatarsal in medial and lateral views. (*e*,*f*) Right fourth metatarsal in medial and lateral views. (*g*,*h*) Right fifth metatarsal in medial and lateral views. (*i*,*j*) Left first metatarsal in dorsal and ventral views. (*k*,*l*) Left fifth metatarsal in medial and lateral views. (*m*,*n*) Proximal phalanx of uncertain position in dorsal and ventral views. (*o*,*p*) Middle phalanx of uncertain position in dorsal and ventral views. (*q*,*r*) Middle phalanx of uncertain position in dorsal and ventral views.

Metatarsals (figure 14*a*–*l*; electronic supplementary material, table S20): both first metatarsals, the right third metatarsal, the fourth metatarsal and both fifth metatarsals are preserved.

The body of the first metatarsal is laterally semicircular in cross section. Its proximal facet is damaged, but it appears to have been a polygon. There is a shallow ventral groove in the proximal portion.

The third metatarsal is relatively short and robust and circular in cross section. Its proximal facet for the tarsal(s) is I-shaped. There are two upper and lower facets on each lateral side near the proximal end for the articulation with the neighbouring metatarsal, which connects to the facet for the ectocuneiform. The distal facet is nearly trapezoidal in outline, though it is partially damaged.

The fourth metatarsal is preserved only in its proximal half, which has a laterally narrow facet for the cuboid and neighbouring metatarsals. In one side, the facets are J-shaped for the fifth metatarsal and C-shaped for the third metatarsal.

The fifth metatarsal is also preserved only in its proximal half. Its proximal facet is oblique to fit the cuboid laterally, and the surface is C-shaped (the C opens ventrally). The body is a rounded rectangle in cross section. There is a small process on the dorsal surface of the proximal end. The distal facet is a rounded triangle in shape.

Phalanges of pes (figure 14*m*–*r*; electronic supplementary material, table S21): one proximal and two middle phalanges are preserved. It is, however, unclear if they came from the left or right foot.

The first proximal phalanx preserved only in its distal portion of the body is semicircular in cross section and is largest among the preserved phalanges. Two middle phalanges are relatively short and dorsoventrally flat. Their distal ends are rectangular with rounded corners, whereas the proximal ends are laterally wide.

## 5. Phylogenetic analysis

**Figure 15.**
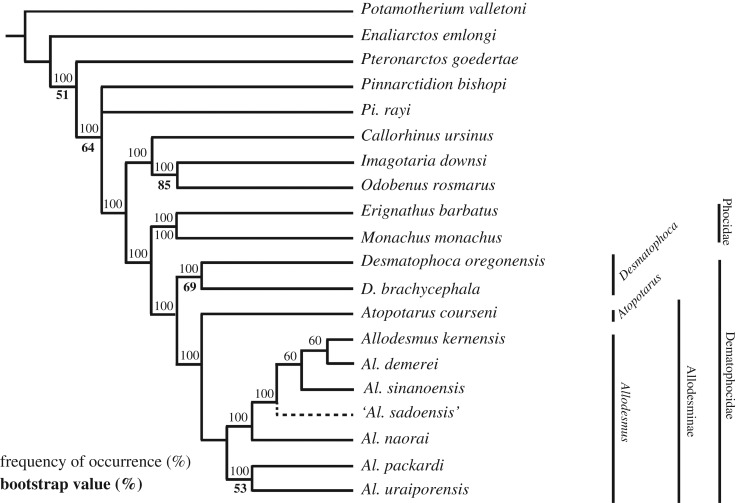
The 50% majority consensus tree of the ten MPTs obtained in this study.

Our analysis of 20 taxa with 100 characters resulted in 10 most parsimonious trees (tree length = 239, consistency index (CI) = 0.6067, retention index (RI) = 0.6908), and their 50% majority-rule consensus is shown in figure 15. As suggested by previous studies (e.g. [13,14,16,17]), the monophyly of Phocoidea (i.e. Desmatophocidae + Phocidae) is recognized, and the clade Desmatophocidae is characterized by five unequivocal synapomorphies: the absence of antorbital process (character 18 : 3), the retracted anterior opening of the carotid canal (character 29 : 1), the long and slender I3 (character 58 : 1), lateral incisors greater in size than medial incisors and medial incisors placed posteromedial to lateral incisor (character 60 : 1), single-rooted M^1^ (character 83 : 2). In addition, three equivocal synapomorphies provide additional support: the long anteroventral process of the jugal (character 11 : 1), the narrow interorbital bar with narrow sides (character 21 : 1), and the paroccipital process that is enlarged posteriorly but still separate from mastoid process (character 40 : 1).

The Desmatophocidae are composed of two subfamilies: the Desmatophocinae consisting of the single genus *Desmatophoca* and the Allodesminae consisting of two genera *Atopotarus* and *Allodesmus*.

The monophyly of the Allodesminae is supported by eight unequivocal synapomorphies: the large and dorsoventrally elliptical anterior narial opening with thick margin (character 3 : 1), the large and broad hamular process (character 15 : 1), the long and shallow glenoid fossa (character 31 : 1), the posteroventral terminus of mandibular symphysis that is relatively horizontal and posterior to the level of P2 (character 48 : 1), the sinuous ventral border of the mandible (character 51 : 1), the anterodorsally directed mandibular foramen (character 54 : 1), bulbous crowns of postcanines (character 68 : 1), and root lobes of postcanine teeth that are inflated and wider than crowns (character 69 : 1). The clade is moderately supported by the bootstrap value of 66%.

*Atopotarus* presently consists of the single species, *Atopotarus courseni*, and it is distinguished from all species of *Allodesmus* by one autapomorphy: the absence of M2 (character 87 : 1). In addition, it retains plesiomorphic characters such as the absence of the prenarial shelf (character 1 : 0) and double-rooted premolars (characters 78 : 0, 79 : 0 and 80 : 0) and molars (character 84 : 0). In this regard, *At. courseni* is thought to be a more basal taxon than *Allodesmus*.

The monophyly of the genus *Allodesmus* consisting of six species (see below, Discussion, §6.1) including *Al. uraiporensis* sp. nov. is supported by the following unequivocal synapomorphy: the presence of the prenarial shelf (character 1 : 1/2). Besides, there are three equivocal synapomorphies supporting this clade: the presence of the facet for the tympanohyal within the hyoid fossa (character 26 : 1), weak and smooth lingual cingulum of P1–2 (character 77 : 2), and the single-rooted P4 (character 82 : 2).

The ‘broad head' subgroup (*Allodesmus naorai* + *Al. packardi*) *sensu* Kohno [13] was not recovered in our analysis, but the ‘long head' subgroup *sensu* Kohno [13] consisting of *Al. kernensis*, *Al. demerei* and *Al. sinanoensis* was recovered as a sister clade of *Al. naorai*. The monophyly of ‘long head' subgroup is supported by following three unequivocal synapomorphies: the well-developed prenarial shelf with anterolateral expansion (character 1 : 2), the transversely and longitudinally arched palate (character 12 : 2), and the slightly divergent palatal margins of the maxilla (character 13 : 0).

*Allodesmus uraiporensis* sp. nov. and *Al. packardi* form a clade which has the rounded infraorbital foramen (character 9 : 0) as an unequivocal synapomorphy and is supported by the bootstrap value of 53%. This clade is the sister group of the clade consisting of the ‘long head' taxa and *Al. naorai*.

*Allodesmus uraiporensis* sp. nov. has at least three unique characters not known in any other species of the genus: the palatine fissure (incisive foramen) that is located anterior to the canine (character 7 : 1), an anteriorly located supraorbital process of the frontal (character 20 : 0), and the developed peroneal tubercle of the calcaneum (character 98 : 1). Most of those characters that differentiate *Al. uraiporensis* from other species are outside the individual variation of at least the extant taxa of otariid pinnipeds [34]. In this regard, *Al. uraiporensis* sp. nov. is a distinctive species, and the establishment of a new taxon is warranted.

## 6. Discussion

### 6.1. Generic assignments of allodesmine species

In previous studies, the classification of the Allodesminae or the genus *Allodesmus* has been controversial in regard to the recognition of different genera and species. In this section, we review the historical background of disputed taxa, and discuss their synonymy.

Barnes & Hirota [12] recognized eight species within four genera, *Atopotarus*, *Brachyallodesmus*, *Megagomphos* and *Allodesmus*, in their non-cladistic study of this subfamily. On the other hand, Kohno [13] recognized only five species within the single genus *Allodesmus* in his cladistic analysis, although interspecific relationships among the five species were almost the same as those of Barnes & Hirota [12]. Deméré & Berta ([16] p. 40) recognized only four species within the single genus *Allodesmus* because of the incompleteness of material for a few species recognized by Barnes & Hirota [12] and Kohno [13]. They also suspected that Barnes & Hirota [12], who recognized eight species of ‘allodesmines’, had over-split the subfamily. In the latest study, Boessenecker & Churchill [14] also indicated that ‘allodesmines’ had a problem of over-split, and they supressed those genera and species into two genera (i.e. *Atopotarus* and *Allodesmus*) and only six species in *Allodesmus* (i.e. *Al. kernensis*, *Al. sinanoensis*, *Al. sadoensis*, *Al. packardi*, *Al. naorai* and *Al demerei*).

Barnes & Hirota [12] established a new genus *Megagomphos* for *Allodesmus sinanoensis*, but Kohno [13] and Boessenecker & Churchill [14] questioned this nomenclatural act, because the absence of prenarial shelf recognized by Barnes & Hirota [12] is due to post-mortem compression of the specimen. We also consider a separate generic assignment for *A. sinanoensis* as not necessary. Barnes & Hirota [12] also erected another genus *Brachyallodesmus* for *Allodesmus packardi*, because they considered the character combination of this species to be far different from that of *Al*. *kernensis*. The numbers of characters for establishing separate genera for *Al. sinanoensis* and *Al. packardi* are not sufficient, whereas these two and remaining species of *Allodesmus* form a clade. Therefore, we follow Deméré & Berta [16] and Boessenecker & Churchill [14] in regarding the two genera as junior synonyms of *Allodesmus*.

Two large species of *Allodesmus*, *Al. megallos* and *Al. sadoensis*, were described from the middle Miocene of central Honshu, Japan also by Barnes & Hirota [12]. We consider them as junior synonyms of *Al. sinanoensis* (Nagao, 1941) for the reasons explained below, as this species name has priority over the other two.

The holotype of *Allodesmus megallos* is a large rostrum without distortion and discovered in the geological unit that yielded the holotype of *Al. sinanoensis*. It was initially identified as *Al*. *kernensis* by Hirota *et al*. [35] because of the presence of anteriorly well-expanded prenarial shelf, but later it was re-described as the holotype of the new species *Al. megallos* by Barnes & Hirota [12] because of its exceptionally large size. We follow Kohno [13] and Boessenecker & Churchill [14] in considering this species a junior synonym of *Al. sinanoensis* because the morphology of the rostrum closely matches between the holotypes of two species, although the former is strongly distorted. The preserved portion of the holotype of *Al*. *sinanoensis* exhibits an anterolaterally expanded prenarial shelf, but Barnes & Hirota [12] did not recognize it because this part of the holotype skull has been strongly laterally compressed and distorted so that the prenarial shelf is not obvious.

*Allodesmus sadoensis* is represented by a single specimen from the middle Miocene Tsurushi Formation in Sado City (=Sado Island), Niigata Prefecture. The holotype is composed of a nearly complete cranium, which exhibits an anteriorly broad rostrum with slender upper canines, large and bulbous cheek teeth, weak sagittal and lambdoidal crests and fragmentally left and right mandibles. It was originally identified as *Al*. *kernensis* by Hirota *et al*. [35] because of the similar cranial morphology. Barnes & Hirota [12] interpreted the holotype cranium of *Al. sadoensis* as belonging to an adult male because of its large size and robustness along with well-abraded cheek teeth, and the undeveloped sagittal crest and unfused sutures were interpreted as paedomorphic.

Boessenecker & Churchill [14] suggested that *Al. sadoensis* was distinguished from *Al. sinanoensis* by its smaller body size and by having a proportionally smaller I^3^, proportionally larger postcanines and the absence of postcanine diastemata. However, these characters are ontogenetically different and sexually dimorphic for instance among species of oatariid or odobenid pinnipeds. In fact, the incomplete suture closures on the holotype skull of *Allodemus sadoensis* indicate that this specimen belongs to a young-adult individual, and a combination of the characters such as the relatively slender canine and less developed sagittal crest are characteristics of female individuals, whereas the degree of cheek tooth abrasion is individually variable. In our analysis, we could not find any supporting characters to differentiate *Al. sinanoensis* and *Al. sadoensis* except also for the above characters mentioned by Boessenecker & Churchill [14]. Furthermore, the holotype of *Al*. *sadoensis* shares quite a few synapomorphic characters seen in *Al. sinanoensis* such as the well-developed and laterally expanded prenarial shelf, the transversely and longitudinally arched palate. Therefore, the holotype of *Al. sadoensis* probably represents a female individual of *Al. sinanoensis* because of its more slender canine and less-developed sagittal crest compared to the holotype and referred rostra of *Al*. *sinanoensis* [36].

Kohno [13] recognized a clade ‘long head' subgroup (*Al. kernensis* + *Al. sinanoensis*) as described above. In our analysis, *Allodesmus demerei* is the sister taxon of *Al. kernensis* and shares the characters of this subgroup such as the presence of anterolaterally well-developed prenarial shelf (character 1 : 2), the transversely and longitudinally arched palate (character 12 : 2), as well as the slightly divergent palatal margins of the maxilla (character 13 : 0). Therefore, the long head subgroup is still recognizable including *Al*. *demerei*.

**Figure 16.**
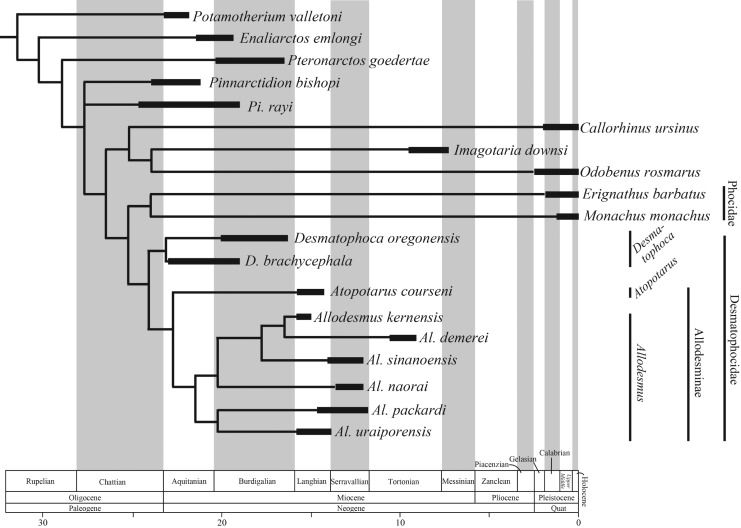
Time calibrated phylogeny of the Allodesminae and related taxa. *Al. sadoensis* is omitted due to possible synonymy (see Discussion). Age ranges of species based on [14,37]; time scale based on [38].

In summary, we consider *Brachyallodesmus* and *Megagomphos* are junior synonyms of *Allodesmus*, and the Allodesminae include only two genera, *Atopotarus* and *Allodesmus*, the former of which is a monospecific genus. As for the genus *Allodesmus*, *Al. megallos* and *Al. sadoensis* are junior synonyms of *Al. sinanoensis*, and only six species; i.e. *Al. kernensis*, *Al. sinanoensis*, *Al. packardi*, *Al. naorai*, *Al. demerei* and *Al. uraoporensis* sp. nov., are recognized (figure 16).

### 6.2. Palaeobiogeographical significance of *Allodesmus uraiporensis* sp. nov.

The early middle Miocene (*ca* 16.3–13.5 Ma [22]) occurrence of the holotype of *Allodesmus uraiporensis* represents the oldest record of this genus in the western North Pacific. *Allodesmus uraiporensis* has relatively primitive characters such as smaller prenarial shelf on the rostrum, relatively anteriorly located supraorbital process of the frontal, smaller molars rather than premolars, and relatively small in overall size, suggesting that the species existed at the time close to the diversification of allodesmine genera and species in the North Pacific realm.

The centre of diversification and dispersal of allodesmines has been thought to be in the eastern North Pacific along the west coast of North America because of the occurrence of their sister taxon *Desmatophoca* and their basal taxon *Pinnarctidion* in the west coast of North America (e.g. [17,39]). The species belonging to *Allodesmus* have been thought to have diversified in the eastern North Pacific and spread out into the western North Pacific almost simultaneously at the time of their diversification. The locality of *Al. uraiporensis* is the northernmost among the Japanese *Allodesmus* [13,22]. According to Ogasawara [40], the location of Hokkaido in the early middle Miocene was the cool temperate sea zone of Horikoshi [41]. The emergence of *Al. uraiporensis* in the early middle Miocene of the western North Pacific might have represented the first arrival of the genus from the eastern North Pacific at the time of their diversification in that region during the middle Miocene Climatic Optimum [42]. The presence of *Allodesmus* in the western North Pacific at the same time as their diversification in the eastern North Pacific suggests a rapid westward diversification of the genus from the eastern North Pacific during that period. Accordingly, *Al. uraiporensis* sp. nov. might have been a forerunner of allodesmines in the western North Pacific. Meanwhile, the occurrence of *Atopotarus* sp. from lower middle Miocene in Hokkaido, Japan [43,44] suggest that the allodesmine diversity might have already reached a certain level by the middle Miocene. Further sampling of lower Miocene marine rocks in Japan is necessary to clarify the timing of geographical diversification of the Allodesminae.

## 7. Conclusion

A nearly complete skeleton containing 92 bones including a cranium, vertebrae, ribs, sternebrae, fore- and hindlimbs from the middle Miocene Okoppezawa Formation (*ca* 16.3–13.9 Ma) is described as the holotype of *Allodesmus uraiporensis* sp. nov. Our phylogenetic analysis suggests that the subfamily Allodesminae is represented by at least by two genera, *Atopotarus* and *Allodesmus*, and at least seven species of the two genera are considered valid. The genus *Allodesmus* consists of six species: *Al. kernensis*, *Al. sinanoensis*, *Al. naorai*, *Al. packardi, Al. demerei* and *Al. uraiporensis* sp. nov. Among them, the ‘long head' subgroup is still recognizable, and it consists of *Al*. *kernensis*, *Al*. *sinanoensis* and *Al. demerei*. On the other hand, the ‘broad head' subgroup is not a robust group and consists of *Al. packardi* and *Al. uraiporensis*. *Allodesmus naorai* is still known only by the incomplete holotype skull, and it is not included in the ‘broad head' subgroup; instead it is recognized as a basal or a sister taxon of the ‘long head' subgroup. *Allodesmus uraiporesis* sp. nov. is recognized as a distinctive new species characterized by two autapomorphies: palatine fissure (incisive foramen) that is located anterior to the canine and well-developed peroneal tubercle of the calcaneum. *Al. uraiporensis* is the oldest and the northernmost record of the genus in the western North Pacific, and suggests either a rapid westward diversification of the genus from the eastern North Pacific or the allodesmine diversity by then was higher than previously perceived.

## Supplementary Material

Electronic supplementary

## Supplementary Material

Data matrix in nexus format

## Data accessibility

Additional data are available as the electronic supplementary materials (Supplementary materials; Data matrix). This study and the nomenclatural acts it contains have been registered in Zoobank. The LSID for this publication is: urn:lsid:zoobank.org:pub:EFF71195-756A-4581-B73F-D5773622CF74.
