# Supplementary material for: A new Miocene pinniped Allodesmus (Mammalia: Carnivora) from Hokkaido, northern Japan
Source: R Soc Open Sci. 2018 May 16;5(5):172440. doi: 10.1098/rsos.172440 (PMC5990790; doi:10.1098/rsos.172440)
Supplement: Electronic supplementary [file rsos172440supp1.docx]

Supplementary Material

Tonomori W, Sawamura H, Sato T and Kohno N. A new Miocene pinniped *Allodesmus* (Mammalia: Caranivora) from Hokkaido, northern Japan. *Royal Society Open Science*

Contents

Table S1-21

List of examined materials

Morphological characters

Table S1. Measurements (mm) of cranium of the holotype (AMP25) of *Allodesmus uraiporensis*. “+” indicates estimated measurements; “*” indicates estimated transverse measurements that are half-cranium measurements multiplied by two; “a” indicates measurements of alveolus.

| measurement regions |  |
| --- | --- |
| Zygomatic width | 200.0*+ |
| Length of tooth row, C to M2 | 120.1 |
| Length of tooth row, P1 to M2 | 92.7 |
| Greatest width of anterior nares | 36.2 |
| Width of rostrum across canines | 96.0* |
| Width between infraorbital foramina | 81.5*+ |
| Width acrosse greatest interobital constriction | 24.0*+ |
| Width across supraorbital processes | 27.6*+ |
| Width of palate between anterior root of P2 | 67.8* |
| Width of palate between anterior root of P4 | 74.0* |
| Transverse diameter of infraorbital foramen | 12.1 |
| Vertical diameter of infraorbital foramen | 13.5 |
| C anteroposterior diameter of root | 30.4a |
| C transverse diameter of root | 14.3a |
| P1 anteroposterior diameter of root | 20.8a |
| P1 transverse diameter of root | 9.5a |
| P2 anteroposterior diameter of root | 14.4a |
| P2 transverse diameter of root | 14.6a |
| P3 anteroposterior diameter of root | 10.5a |
| P3 transverse diameter of root | 12.7a |
| P4 anteroposterior diameter of root | 10.8a |
| P4 transverse diameter of root | 9.3a |
| M1 anteroposterior diameter of root | 7.6a |
| M1 transverse diameter of root | 7.3a |
| M2 anteroposterior diameter of root | 7.6a |
| M2 transverse diameter of root | 5.2a |
| C-P1 septum length | 2.3 |
| P1-P2 septum length | 2.8 |
| P2-P3 septum length | 2.3 |
| P3-P4 septum length | 7.3 |
| P4-M1septum length | 9.5 |
| M1-M2 septum length | 2.7 |

Table S2. Measurements (mm) of cervical vertebra of the holotype (AMP25) of *Allodesmus uraiporensis*. “+” indicates estimated measurements.

| measurement regions | 5th | 6th | 7th |
| --- | --- | --- | --- |
| Greatest height, tip of neural spine to inferior face of centrum | 101.0 | - | 128.0 |
| Distance across vertebra between tips of transverse processes | - | - | 138.0+ |
| Length of centrum | - | 70.0 | - |
| Least anteroposterior diameter of neural arch | 14.0+ | - | 16.0 |
| Distance between outside margins of prezygapophyses | - | - | - |
| Distance between outside margins of postzygapophyses | 72.0 | 91.0 | 98.0 |
| Tip of prezygapophysis to tip of postzygapophysis | - | 84.0 | - |

Table S3. Measurements (mm) of thoracic vertebra of the holotype (AMP25) of *Allodesmus uraiporensis*. “+” indicates estimated measurements.

| measurement regions | 6th | 7th | 8th |
| --- | --- | --- | --- |
| Greatest height, tip of neural spine to inferior face of centrum | - | - | - |
| Distance across vertebra between tips of transverse processes | - | - | - |
| Length of centrum | - | 51.0 | 50.0 |
| Least anteroposterior diameter of neural arch | 13.0 | - | - |
| Distance between outside margins of prezygapophyses (mammillary process) | - | - | - |
| Distance between outside margins of postzygapophyses | - | - | - |
| Tip of prezygapophysis to tip of postzygapophysis | - | - | - |

| measurement regions |  |  |  |
| --- | --- | --- | --- |
| Greatest height, tip of neural spine to inferior face of centrum | 11th | 12th | 13th |
| Distance across vertebra between tips of transverse processes | 100.0 | 95.0 | - |
| Length of centrum | - | - | - |
| Least anteroposterior diameter of neural arch | 51.0 | 53.0 | 51.0 |
| Distance between outside margins of prezygapophyses (mammillary process) | 24.0 | 29.0 | - |
| Distance between outside margins of postzygapophyses | - | 71.0+ | 73.0 |
| Tip of prezygapophysis to tip of postzygapophysis | - | 42.0+ | 45.0 |
|  | - | 83.0+ | 85.0 |

| measurement regions | 14th | 15th |
| --- | --- | --- |
| Greatest height, tip of neural spine to inferior face of centrum | - | 107.0 |
| Distance across vertebra between tips of transverse processes | - | - |
| Length of centrum | 51.0 | 55.0 |
| Least anteroposterior diameter of neural arch | - | 39 |
| Distance between outside margins of prezygapophyses (mammillary process) | 76.0 | 75.0 |
| Distance between outside margins of postzygapophyses | 41.0 | 42.0 |
| Tip of prezygapophysis to tip of postzygapophysis | 82.0 | 85.0 |

Table S4. Measurements (mm) of lumbar vertebra of the holotype (AMP25) of *Allodesmus uraiporensis*.

| measurement regions | 1st | 2nd | 3rd |
| --- | --- | --- | --- |
| Greatest height0 tip of neural spine to inferior face of centrum | 112.0 | - | 114.0 |
| Distance across vertebra between tips of transverse processes | - | - | - |
| Length of centrum | - | 54.0 | 55.0 |
| Least anteroposterior diameter of neural arch | 32.0 | - | 24.0 |
| Distance between outside margins of prezygapophyses (mammillary process) | 77.0 | - | - |
| Distance between outside margins of postzygapophyses | - | - | 47.0 |
| Tip of prezygapophysis to tip of postzygapophysis | - | 88.0 | 88.0 |

| measurement regions | 4th |
| --- | --- |
| Greatest height, tip of neural spine to inferior face of centrum | - |
| Distance across vertebra between tips of transverse processes | - |
| Length of centrum | 53.0 |
| Least anteroposterior diameter of neural arch | - |
| Distance between outside margins of prezygapophyses (mammillary process) | - |
| Distance between outside margins of postzygapophyses | - |
| Tip of prezygapophysis to tip of postzygapophysis | - |

Table S5. Measurements (mm) of sacrum of the holotype (AMP25) of *Allodesmus uraiporensis*.

| measurement regions |  |
| --- | --- |
| Greatest length on the ventral side | 174 |
| Greatest width | 137 |
| Greatest height(vertically), tip of neural spine to inferior face of centrum | 98 |
| Distance between outside margins of prezygapophyses | 62 |

Table S6. Measurements (mm) of caudal vertebrae of the holotype (AMP25) of *Allodesmus uraiporensis*.

| measurement regions | 1st | 2nd | 3rd |
| --- | --- | --- | --- |
| Greatest height (vertically), tip of neural spine to inferior face of centrum | 57 | 50 | 39 |
| Greatest width | 56 | 45 | 47 |
| Distance between outside margins of prezygapophyses | 21 | 41 | 31 |
| Distance between outside margins of postzygapophyses | 40 | - | - |
|  |  |  |  |
| measurement regions | 4th | 5th | 6th |
| Greatest height (vertically), tip of neural spine to inferior face of centrum | 33 | 26 | 25 |
| Greatest width | 43 | 41 | 33 |
| Distance between outside margins of prezygapophyses | 25 | 21 | 18 |
| Distance between outside margins of postzygapophyses | - | - | - |
|  |  |  |  |

| measurement regions | 7th | 8th |
| --- | --- | --- |
| Greatest height (vertically), tip of neural spine to inferior face of centrum | 24 | 22 |
| Greatest width | 30 | 27 |
| Distance between outside margins of prezygapophyses | 17 | - |
| Distance between outside margins of postzygapophyses | - | - |

Table S7. Measurements (mm) of phalanx of manus of uncertain position of the holotype (AMP25) of *Allodesmus uraiporensis*.

| measurement regions |  |
| --- | --- |
| Greatest length | 55.5 |
| (Greatest) width of the proximal end | 21.2 |
| Smallest width of the diaphysis | 14.2 |
| (Greatest) width of the distal end | 23.1 |

Table S8. Measurements (mm) of pelvis of the holotype (AMP25) of *Allodesmus uraiporensis*. “+” indicates estimated measurements.

| measurement regions | R | L |
| --- | --- | --- |
| Greatest length | - | 344.0 |
| Anterior margin of acetabulum to anterior margin of ilium | 53.0 | 52.0 |
| Posterior margin of acetabulum to posterior margin of ischium | 54.0 | 56.0 |
| Antero-posterior diameter of obtulator foramen | 109.0+ | 121.0 |
| Symphysis of pubis to tuberosity of ischium | - | 82.0 |

Table S9. Measurements (mm) of femur of the holotype (AMP25) of *Allodesmus uraiporensis*. “R” indicates right. “L” indicates left.

| measurement regions | R | L |
| --- | --- | --- |
| Greatest length | - | 194.0 |
| Greatest transverse diameter of proximal end (head to greater trochanter) | 94.0 | 94.0 |
| Extero-internal diameter of shaft at narrowest point | 45.0 | 46.0 |
| Greatest transverse diameter of distal end (medial to lateral condyle) | - | 94.0 |
| Greatest diameter of head | 42.0 | 43.0 |
| Greatest transverse diameter of medial condyle | - | 31.0 |
| Greatest transverse diameter of lateral condyle | 42.0 | 42.0 |
| width of intercondyloid fossa at narrowest point | 18.0 | 19.0 |
| width of distal end of shaft | 63.0 | 65.0 |
| width shaft at proximal end | 46.0 | 48.0 |

Table S10. Measurements (mm) of patella of the holotype (AMP25) of *Allodesmus uraiporensis*. “R” indicates right. “L” indicates left.

| measurement regions | R | L |
| --- | --- | --- |
| Greatest length | 62.0 | 60.0 |
| Greatest width | 43.0 | 43.0 |

Table S11. Measurements (mm) of tibia of the holotype (AMP25) of *Allodesmus uraiporensis*. “+” indicates estimated measurements. “R” indicates right. “L” indicates left.

| measurement regions | R | L |
| --- | --- | --- |
| Greatest length | - | 315.0 |
| Greatest diameter of shaft at distal end | - | 46.0 |
| Greatest diameter of shaft at proximal end | 37.0+ | 43.0 |
| Transverse diameter of shaft at narrowest point | - | 29.0 |
| Greatest diameter of proximal epiphysis | - | - |

Table S12. Measurements (mm) of fibula of the holotype (AMP25) of *Allodesmus uraiporensis*. “R” indicates right. “L” indicates left.

| measurement regions | R | L |
| --- | --- | --- |
| Greatest length | 287.0 | 290.0 |
| Greatest diameter of shaft at proximal end | 23.0 | 23.0 |
| Greatest diameter of shaft at distal end | 26.0 | 27.0 |
| Least postero-internal diameter of shaft | 14.0 | 16.0 |

Table S13. Measurements (mm) of astragalus of the holotype (AMP25) of *Allodesmus uraiporensis*. “R” indicates right. “L” indicates left.

| measurement regions | R | L |
| --- | --- | --- |
| Greatest vertical diameter | - | 53.0 |
| Greatest transverse diameter (head to lateral process) | - | 64.0 |
| Greatest diameter of head | - | 47.0 |
| Greatest dorso-plantar diameter of body | - | 27.0 |

Table S14. Measurements (mm) of calcaneum of the holotype (AMP25) of *Allodesmus uraiporensis*. “R” indicates right. “L” indicates left.

| measurement regions | R | L |
| --- | --- | --- |
| Least transverse diameter of shaft | 29.0 | 29.0 |
| Greatest transverse diameter of cuboid facet | 31.0 | 32.0 |
| Greatest width, medialo-laterally | 68.0 | 66.0 |
| Greatest length, proximo-distally | 96.0 | 96.0 |

Table S15. Measurements (mm) of navicular of the holotype (AMP25) of *Allodesmus uraiporensis*. “R” indicates right. “L” indicates left.

| measurement regions | R | L |
| --- | --- | --- |
| Greatest width | 58.0 | 60.0 |
| Greatest height | 37.0 | 38.0 |
| Height of posterior articular surface | 27.0 | 27.0 |
| Width of posterior articular surface | 48.0 | 48.0 |

Table S16. Measurements (mm) of cuboid of the holotype (AMP25) of *Allodesmus uraiporensis*. “R” indicates right. “L” indicates left.

| measurement regions | R | L |
| --- | --- | --- |
| Greatest width | 36.0 | - |
| Width of posterior articular surface | 20.0 | - |
| Greatest length | 50.0 | - |
| Greatest height | 40.0 | - |

Table S17. Measurements (mm) of ectocuneiform of the holotype (AMP25) of *Allodesmus uraiporensis*. “R” indicates right. “L” indicates left.

| measurement regions | R | L |
| --- | --- | --- |
| Greatest length | 27.0 | 28.0 |
| Greatest width | 23.0 | 22.0 |
| Greatest height | 47.0 | 47.0 |

Table S18. Measurements (mm) of mesocuneiform of the holotype (AMP25) of *Allodesmus uraiporensis*. “R” indicates right. “L” indicates left.

| measurement regions | R | L |
| --- | --- | --- |
| Greatest length | 22.9 | 22.2 |
| Greatest width | 14.5 | 14.2 |
| Greatest height | 30.3 | 31.9 |

Table S19. Measurements (mm) of entocuneiform of the holotype (AMP25) of *Allodesmus uraiporensis*. “R” indicates right. “L” indicates left.

| measurement regions | R | L |
| --- | --- | --- |
| Greatest length | 44.0 | 43.0 |
| Greatest width | 45.0 | 46.0 |
| Greatest height | 24.0 | 24.0 |

Table S20. Measurements (mm) of metatarsals of the holotype (AMP25) of *Allodesmus uraiporensis*. “R” indicates right. “L” indicates left.

| measurement regions | 1st | | 3rd | | 4th | |
| --- | --- | --- | --- | --- | --- | --- |
|  | R | L | R | L | R | L |
| Greatest length | 112.0 | - | - | 94.0 | - | - |
| (Greatest) width of the proximal end | - | - | - | 18.8 | - | 21.6 |
| (Greatest) height of the proximal end | - | - | - | 36.1 | - | 40.6 |
| Smallest width of the diaphysis | 23.6 | - | - | 13.4 | - | - |
| (Greates) width of the distal end | 30.6 | 27.5 | - | - | - | - |

| measurement regions | 5th | |
| --- | --- | --- |
|  | R | L |
| Greatest length | 119.0 | - |
| (Greatest) width of the proximal end | 24.5 | 27.3 |
| (Greatest) height of the proximal end | 51.3 | 50.3 |
| Smallest width of the diaphysis | 19.2 | - |
| (Greates) width of the distal end | 26.5 | - |

Table S21. Measurements (mm) of phalanx of pes of uncertine of position of the holotype (AMP25) of *Allodesmus uraiporensis*. “R” indicates right. “L” indicates left.

| measurement regions | fig 9Q, R | fig 9S, T | fig 9U, V |
| --- | --- | --- | --- |
| Greatest length | - | 64.4 | 55.7 |
| (Greatest) width of the proximal end | 23.1 | 19.3 | 22.4 |
| Smallest width of the diaphysis | - | 13.6 | 14.2 |
| (Greatest) width of the distal end | - | 18.0 | 20.2 |

List of examined materials including cranium, mandibular and postcranium in this study.

Institutional Abbreviations- AMP, Ashoro Museum of Paleontology, Hokkaido, Japan; NMB, Naturhistorisches Museum, Basel, Switzerland; CAS, California Academy of Sciences, California, USA; LACM, Natural History Museum of Los Angeles County, California, USA; MSFM, Matsumoto Shiga Fossil Museum, Nagano, Japan; NMJH, National Museum of Japanese History, Chiba, Japan; NMNS, National Museum of Nature and Science, Ibaraki, Japan; SICC: Sado Island Community Center, Sado, Niigata, Japan; UCMP: University of California Museum of Paleontology, Berkeley, California, USA; USNM, National Museum of Natural History and Culture, Smithsonian Institution, Washington D. C., USA.

Fossil, Outgroup Taxa

*Potamotherium valletoni*―NMNS-PV 20548 (brain cast of NMB-SAU 2280), NMNS-PV 20549 (brain cast of NMB-SAU 2281), NMNS- PV -20762 (skull), NMNS-PV 20763 (left mandible), NMNS-PV 20766 (axis), NMNS-PV 20832 (left humerus), NMNS-PV 20834 (left radius), NMNS-PV 20839 (left first metacarpus), NMNS-PV 20876 (left astragalus), NMNS-PV 20878 (left calcaneum).

*Enaliarctos emlongi*―USNM 250345 (holotype)

*Pteronarctos goedertae*― LACM 123883 (holotype)

*Pinnarctidion bishopi*―UCMP 86334 (holotype)

*Pinnarctidion rayi*―USNM 314325 (holotype), USNM 250321, USNM 335383

Extant, Ingroup Taxa

*Callorhinus ursius*―NMNS M29642, NMNS M46874, USNM 12749, USNM 21333

*Odobenus rosmarus*―NMNS M27862, USNM 500254, USNM 504669

*Erignathus barbatus*―USNM 500249, USNM 500250, NMNS-POM 165

*Monachus monachus*―USNM 219059

Fossil, Ingroup Taxa

*Imagotaria downsi*― NMNS-PV 15077 (cast of the holotype)

*Desmatophoca oregonensis*―NMNS-PV 15076 (cast of the holotype)

*Desmatophoca brachycephala*―LACM 120199 (holotype)

*Atopotarus courseni*―LACM 1376 (holotype)

*Allodesmus kernensis*― LACM 4320 (holotype of *Allodesmus* *kelloggi*), LACM 4956, UCMP 81708 (holotype of *Allodesmus* *gracile*).

*Allodesmus sinanoensis*―MSFM 00006 (holotype), MSFM 00002 (holotype of *Allodesmus* *megallos*), SICC 0001 (holotype of *Allodesmus* *sadoensis*, new synonimization)

*Allodesmus naorai*―NMJH A-636-1-1-16 (holotype, formerly N-001)

*Allodesmus packardi*―CAS 4371A (holotype)

*Allodesmus uraiporensis*―AMP 25 (holotype)

Morphological characters

Cranium

1. Prenarial shelf: 0=absent. 1=developed. 2=well developed with lateral expansion

2. Premaxilla: rostral process. 0= absent. 1= present.

3. Anterior narial opening. 0= large, thin margin, and rounded. 1= large, thick

margin, and dorsoventrally elliptical.

4. Premaxilla: ascending process along nasal. 0= overlap with nasal long, little contact of nasal and maxilla. 1= short overlap, no contact of ascending premaxilla and frontal. 2= very short overlap.

5. Nasal: posterior end. 0= converging. 1= parallel with transversely straight frontal/maxillary suture. 2= parallel with broad V-shaped frontal/maxillary suture. 3= diverging. 4= penetrating frontal.

6. Palatine fissures (incisive foramina). 0= distinct pair. 1= foramina coalesced. 2= trilobed pits. 3= single pit or reduced.

7. Palatine fissures (incisive foramina). 0= aligned with canine. 1= located anterior to canine. 2=absent. New character.

8. Infraorbital foramen. 0= small. 1= enlarged. 2=greatly enlarged

9. Infraorbital foramen. 0= rounded. 1= transversally oval. 2=dorsaroventrally oval. New character

10. Ventral tuberosity of zygomatic root. 0= absent. 1= present.

11. Jugal: anteroventral process. 0= moderate. 1= long, reaching M1. 2= absent.

12. Palate. 0= flat. 1= arched transversely. 2= arched transversely and longitudinally.

13. Maxilla: palatal margins. 0= slightly divergent. 1= parallel. 2= posteriorly very wide.

14. Palatine. 0= small. 1= long and posterolaterally expanded. 2= telescoped and underlying alisphenoid.

15. Pterygoid: hamular process. 0= small and narrow. 1= large and broad.

16. Pterygoid strut. 0= slender. 1= dorsoventrally thin and laterally projected. 2= dorsoventrally thick and laterally broad. 3= laterally thin and rolled.

17. Orbit: fossa muscularis. 0= present. 1= absent.

18. Orbit: antorbital process. 0= small ridge on frontal. 1= prominent on frontal and maxilla. 2= prominent on maxilla only. 3= absent.

19. Frontal: supraorbital process. 0= small bump. 1= absent. 2= large and shelf like.

20. Frontal: supraorbital process. 0=anteriorly located. 1=posteriorly located. 2=absent. New character.

21. Frontal: interorbital bar. 0= moderate in width, broader than intertemporal region. 1= narrow, parallel sided. 2=narrow, narrower than intertemporal region. 3= very broad and short.

22. Jugal: postorbital process. 0= small. 1= dorsally projecting.

23. Zygomatic process. 0= long and slender. 1= short and slender. 2= dorsoventrally deep. 3= exaggeratedly deep.

24. Orbital vacuity. 0= absent. 1= present/anteriorly positioned. 2= present/posteriorly positioned.

25. Orbit: optical foramen and orbitosphenoid. 0= plate-like. 1= funnel shaped.

26. Facet for tympanohyal within hyoid fossa. 0=absent. 1=present.

27. Internal auditory meatus. 0= rounded. 1= bilobed. 2= canals for cranial nerves VII and VIII separated.

28. Epitympanic recess. 0= small. 1= large. 2= very large.

29. Anterior opening of carotid canal. 0= proportionate. 1= retracted.

30. Squamosal fossa on zygomatic root. 0= present. 1= reduced.

31. Glenoid fossa. 0=long and deep. 1=long and shallow. 2=short and shallow.

32. Squamosal: transverse ridge. 0= absent. 1= present.

33. Pseudosylvian sulcus. 0= present, deep. 1= reduced or absent.

34. Bony tentorium. 0= far from petrosal. 1= appressed to petrosal. 2= reduced.

35. Basioccipital. 0= narrow and parallel sided. 1= broad and pentagonal.

36. Inferior petrosal sinus. 0=present. 1=absent.

37. Posterior lacerate foramen. 0= round. 1= transversely expanded. 2= fissure.

38. Lambdoidal crest. 0= crestlike, posterodorsally directed. 1= flattened, anterodorsally directed.

39. Mastoid process. 0= small. 1= large. 2= very large as widest part of skull. 3= pachyostotic.

40. Paroccipital process. 0= small and separated from mastoid process. 1= enlarged posteriorly but still separate from mastoid process. 2= moderately sized and joined with mastoid process by a continuous ridge. 3= flattened and plate-like.

Mandible

41. Mandible:mandibular symphysis. 0=separated. 1=fused.

42. Mandible: length of mandibular symphysis. 0=less than 50% of length of horizontal ramus. 1= greater than 50% of length of horizontal ramus.

43. Mandible: genial tuberosity. 0= absent or indistinct. 1= present; developed as small tubercle or process on anterior portion of ramus. 2= present and well developed; extends well below ventral margin of ramus.

44. Mandible: anterior portion of symphyseal region. 0= smooth, compact bone. 1=rugose, vascular bone.

45. Mandible: horizontal ramus. 0= horizontal. 1= upturned.

46. Mandible: mandibular furrow. 0= absent. 1= anterodorsal end of mandibles have a longitudinal furrow anteriorly.

47. Mandible: edentulous mandibular terminus. 0= absent. 1= present.

48. Mandible: posteroventral terminus of mandibular symphysis. 0= relatively vertical, same level or anterior to P_1_ or P_2_. 1= relatively horizontal, posterior to the level of P_2_.

49. Mandible: depth of horizontal ramus. 0= deepest part of horizontal ramus posterior to mandibular symphysis. 1= deepest part of horizontal ramus at posteroventral terminus of symphysis.

50. Mandible: mandibular arch. 0= nearly parallel. 1= sharply divergent.

51. Mandible: ventral border. 0= straight. 1= sinuous.

52. Mandible: enlarged digastric insertion. 0= absent. 1= present.

53. Mandible: mandibular condyle. 0 = at or slightly above level of tooth row. 1 = elevated above tooth row.

54. Mandible: mandibular foramen. 0 = directed anteroventrally. 1 = directed anterodorsally.

55. Mandible: medial shelf of angular process. 0= does not form medial shelf. 1= forms small medial shelf. 2=forms large medial shelf or projection.

56. Mandible: base of coronoid process. 0= narrow (less than 42% of mandible length). 1= broad (greater than or equal to 42% of mandible length).

Dentition

57. Upper incisors: number. 0= three. 1= two. 2= one. 3= incisors absent.

58. Upper I^3^. 0= moderate. 1= long and slender. 2= premolariform. 3= absent.

59. Lower incisors: number. 0= 2 incisors. 1= 1 incisor. 2= incisors absent.

60. Lower incisors: relative size. 0= lower incisors of equal size, form transverse arcade. 1= lateral incisors greater in size than medial incisors, medial incisor placed posteromedial to lateral incisor.

61. C^1^. 0= caniniform with posterior crista. 1=caniniform without posterior crista. 2= tusk like. 3= tusk like with globular osteodentine.

62. C_1_: size compared to C^1^. 0= nearly equal (100-80%) and caniniform. 1= reduced (75-20%) and premolariform. 2= lower canine absence.

63. C_1_: posterior crista. 0= present. 1= absent.

64. C_1_: longitudinal fluting. 0= absent. 1= present.

65. C_1_: root. 0= oval or circular in cross section. 1= bilobate in cross section or prominent longitudinal labial sulcus.

66. C_1_: orientation. 0= not procumbent and vertically oriented. 1= procumbent.

67. Postcanines: tooth enamel. 0= well developed. 1= thin or patchy. 2= enamel absent in postcanine teeth in adults.

68. Postcanines: crowns. 0= laterally compressed. 1= bulbous.

69. Postcanines: root lobes. 0= root lobes of postcanine teeth of equal or narrower width than crowns. 1= root lobes of postcanine teeth inflated and wider than crowns.

70. Lower postcanines: tooth row length. 0= long (length of tooth row greater than 40% of the mandible length). 1= short (length of tooth row less than or equal to 40% of the mandible length).

71. Lower postcanines: paraconid cusps. 0= present and well developed. 1= absent or reduced.

72. Lower postcanines: lingual cingula. 0= present and smooth. 1= present and rough or crenulated.

73. Lower premolars. 0= P_1-4_ present. 1= P_1-3_ present, P_4_ absent. 2= premolars absent.

74. Lower premolars: hypoconid cusps. 0= present. 1= absent.

75. Lower postcanines: talonid basin. 0= absent. 1= slight concavity or small shelf.

76. Lower postcanines: metaconid. 0= present. 1= reduced. 2= absent.

77. P^1-2^, lingual cingulum. 0= distinct but small. 1= well developed with cuspules. 2= weak and smooth. 3= P^1-2^ absent.

78. P_2_: roots. 0= double rooted or bilobate. 1= single rooted.

79. P_3-4_: roots. 0= double rooted. 1= single rooted.

80. P^3^: roots. 0= double rooted. 1= single and bilobed. 2= single and cylindrical. 3= P3 absent.

81. P^4^, protocone shelf. 0= anteromedially placed. 1= posteromedially placed with small cuspules. 2= reduced or absent.

82. P^4^, roots. 0= triple rooted. 1= double rooted. 2= single rooted. 3= P^4^ absent.

83. M^1^: roots. 0= triple rooted. 1= double rooted. 2= single rooted. 3= M1 absent.

84. M_1_: roots. 0= double rooted. 1= single rooted. 2=M_1_ absent

85. M^2^: roots. 0= double rooted. 1= single rooted. 2= M^2^ absent.

86. M^1-2^: relative size to premolars. 0=small. 1=nearly equal.

87. M_2_. 0= present. 1= absent.

88. Postcanine tooth wear. 0= absent. 1= minor wear on anterior and posterior cusps or apical wear. 2= large wear facets on anterior and posterior edges. 3= heavily worn and polished.

Postcranium

89. Humerus: deltoid tubercle. 0= on pectoral crest. 1= on lateral edge of crest. 2= off crest.

90. Humerus: diameter of distal trochlea. 0= medial lip same diameter (or smaller) as distal capitulum. 1= medial lip diameter greater than distal capitulum. 2=medial lip diameter less than distal capitulum.

91. Radius: distal end. 0= unexpanded. 1= expanded, with small radial process. 2= expanded, with large radial process.

92. Metacarpal I: insertion of pollicle extensor. 0= smooth. 1= pit. 2= rugosity.

93. Scapholunar. 0= no pit for magnum. 1= well-formed pit.

94. Astragalus: medial plantar tuberosity. 0= absent. 1= present. 2= elongated.

95. Astragalus: facet for cuboid . 0=absent. 1= present. New character.

96. Calcaneum: calcaneal tuberosity. 0= straight. 1= medially prominent.

97. Calcaneum: secondary shelf. 0=absent, 1=present.

98. Calcaneum: peroneal tubercle. 0=present. 1=developed. 2=developed and located on a planta. New character.

99. Entocuneiform/mesocuneiform articulation. 0= abutting. 1= overlapping.

100. The spinous process of the axis. 0= axe-like. 1= blade-like.
